# Population Genomics and Phylogeography of a Clonal Bryophyte With Spatially Separated Sexes and Extreme Sex Ratios

**DOI:** 10.3389/fpls.2020.00495

**Published:** 2020-05-08

**Authors:** Marta Alonso-García, Juan Carlos Villarreal A., Kenneth McFarland, Bernard Goffinet

**Affiliations:** ^1^Département de Biologie, Université Laval, Quebec City, QC, Canada; ^2^Smithsonian Tropical Research Institute, Ancón, Panama; ^3^Department of Ecology and Evolutionary Biology, The University of Tennessee, Knoxville, TN, United States; ^4^Ecology and Evolutionary Biology, University of Connecticut, Storrs, CT, United States

**Keywords:** clonality, hornwort, *Nothoceros*, population genomic, sex separation, SNPs, stacks

## Abstract

The southern Appalachian (SA) is one of the most biodiversity−rich areas in North America and has been considered a refugium for many disjunct plant species, from the last glacial period to the present. Our study focuses on the SA clonal hornwort, *Nothoceros aenigmaticus* J. C. Villarreal & K. D. McFarland. This hornwort was described from North Carolina and is widespread in the SA, growing on rocks near or submerged in streams in six and one watersheds of the Tennessee (TR) and Alabama (AR) Rivers, respectively. Males and female populations occur in different watersheds, except in the Little Tennessee (TN) River where an isolated male population exists ca. 48 km upstream from the female populations. The sex ratio of 1:0 seems extreme in each population. In this study, we use nuclear and organellar microsatellites from 250 individuals from six watersheds (seven populations) in the SA region and two populations from Mexico (23 individuals). We, then, selected 86 individuals from seven populations and used genotyping by sequencing to sample over 600 bi-allelic markers. Our results suggest that the SA *N. aenigmaticus* and Mexican plants are a nested within a clade of sexual tropical populations. In the US populations, we confirm an extreme sex ratio and only contiguous US watersheds share genotypes. The phylogenetic analysis of SNP data resolves four clusters: Mexican populations, male plants (Little Pigeon and Pigeon river watersheds) and two clusters of female plants; one from the Little Tennessee and Hiwassee Rivers (TR) and the other from the Ocoee (TR) and Coosa (AR) Rivers. All clusters are highly differentiated (Fst values over 0.9). In addition, our individual assignment analyses and PCAs reflect the phylogenetic results grouping the SA samples in three clades and recovering males and female plants with high genetic differentiation (Fst values between 0.5 and 0.9 using microsatellites and bi-allelic markers). Our results point to Pleistocene events shaping the biogeographical pattern seen in US populations. The extreme sex ratio reflects isolation and highlights the high vulnerability of the populations in the SA.

## Introduction

Skewed sex ratios, dioicy and clonality are prominent features of plant reproductive systems with profound implications on the ecology, evolution and survival of the species (Barrett, 2015; Petry et al., 2016; Scott et al., 2018). In genetically determined sexual systems, the theoretical sex ratio is expected to be close to 1:1 (female: male), if we assume a similar cost to the production of male and female individuals (Fisher, 1930; Scott et al., 2018). However, sex ratios can be significantly skewed (Bisang and Hedenäs, 2005; Sinclair et al., 2012). For example, 61% of 234 species of angiosperms distributed among 61 families display a male-biased ratio (Field et al., 2013). In bryophytes, most of the species studied display a strong female-biased ratio. In nearly 85% of the 103 moss and liverworts species surveyed (Bisang and Hedenäs, 2005), females outnumbered males in natural populations. One striking example is the desert moss, *Syntrichia caninervis* Mitt. In the Mojave Desert only 11 males were found among 700 individuals examined (Stark et al., 2005). The causes and genetic repercussions of such skewed ratios are now being explored (Bisang and Hedenäs, 2005; Bisang et al., 2015, 2017; Baughman et al., 2017; Brzyski et al., 2018).

In contrast to flowering plants, where separate sexes (diploid dioecy) is rare (Renner and Ricklefs, 1995), most bryophytes species display separate male and female plants (haploid dioicy). Nearly 70% of mosses and liverworts and 40% of hornworts species display separate sexes (Wyatt and Anderson, 1984; Villarreal and Renner, 2013; Laenen et al., 2016). In bryophytes, the gametophyte is free-living, and the male gametes are motile, and consequently, sexual reproduction is constrained by the availability of water between the sexes. Although this constraint may be lessened by degrees of dehydration tolerances of the sperm (Shortlidge et al., 2012), or by invertebrates acting as vectors (Cronberg et al., 2006), the rate of sexual reproduction decreases with the distance between sexes, with an upper limit of several meters (Van der Velde et al., 2001), but typically within a few centimeters (Longton, 1997). Proximity of the sexes is therefore imperative for successful sexual reproduction in bryophytes. The widespread occurrence of clonality may exacerbate the distance between sexes with a preferential growth of female plants (e.g., in *Marchantia*; Brzyski et al., 2018), biasing sex ratios and accentuating the difficulty of sexual reproduction. Despite these functional impediments, spatial separation of sexes (SSS) is widespread among all groups of plants (Bierzychudek and Eckhart, 1988; Groen et al., 2010; Brzyski et al., 2018). The evolution of dioicy may be also driven by the benefits of the SSS (Bierzychudek and Eckhart, 1988), as SSS may favor niche partitioning and avoid intersexual competition for example (Bierzychudek and Eckhart, 1988; Rogers and Eppley, 2015).

The female-biased ratio, the widespread dioicy (Bisang and Hedenäs, 2005; Bisang et al., 2017) and the prominent role of clonality in bryophytes may account for the incidence of SSS with over 20 cases reported at a large geographic scale (Schuster, 1983; Stark et al., 2005; Villarreal et al., 2014; Bisang et al., 2015). In fact, the most extreme examples of SSS in plants are found in bryophytes. For instance, the leafy liverwort *Plagiochila corniculata* Dumort. is known only from female plants in North America and all European individuals bear only male sex organs (Schuster, 1983, 1992). Clonality, limited dispersal and SSS may amplify the non-random spatial distribution of genotypes, or spatial genetic structure (SGS). In fact, the type of mating system seems to contribute to SGS, with higher incidence of SGS in dioecious flowering plants (Nazareno et al., 2013; Dering et al., 2016).

Some repercussions of extreme separation of sexes are SGS or reduced sexual reproduction (Barrett, 2015). In addition, in flowering plants, the proliferation of sterility mutations (the “use-it-or-lose-it” hypothesis; Eckert, 2002) and events of local extinction (Hu et al., 2017) are other consequences of the spatial separation of sexes. In bryophytes, the occurrence of such extreme cases of SSS is intriguing and leads to a number of evolutionary questions about the occurrence of sex-specific SGS in bryophytes with SSS or the time populations can be maintained clonally. Given the limited sexual reproduction and the little genetic diversity, will these populations face local extinction? In the liverwort *Marchantia inflexa* Nees & Mont., some of these questions have already been answered: sexes are differentially clumped (males tend to be near male plants in larger patches), suggesting a fine scale trade-off between sexual reproduction and clonality (Brzyski et al., 2018).

**FIGURE 1:**
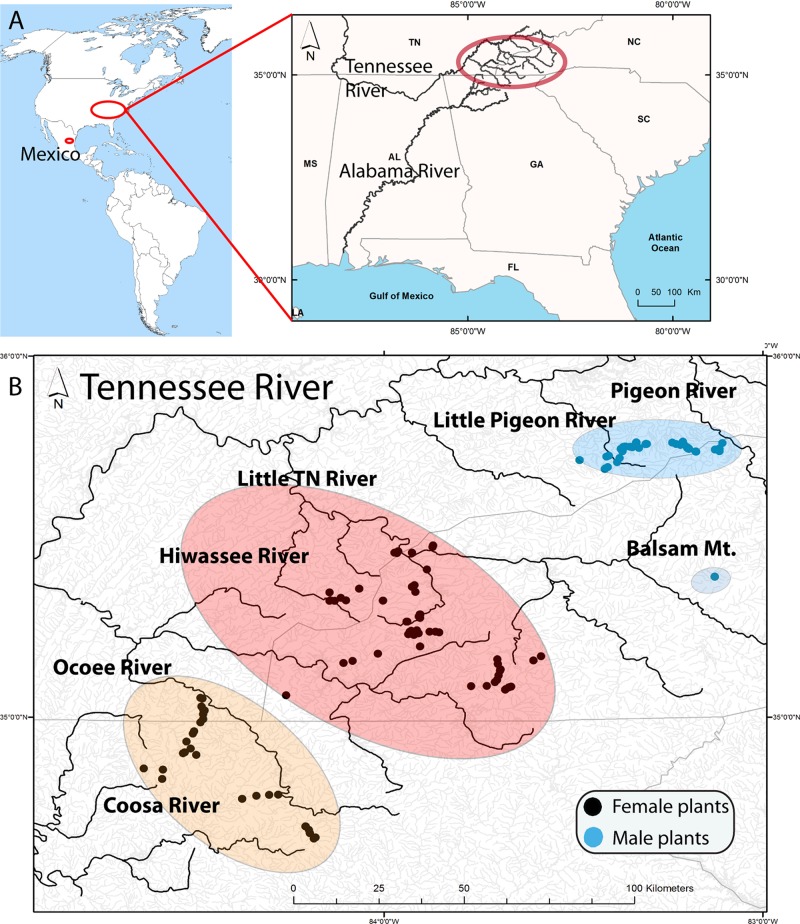
**(A)** Location of samples in America with a map of the Southern United States illustrating the Tennessee River (Mississippi River) and the Coosa River (Alabama River) that drain in the Gulf of Mexico. The upper Tennessee River, where the samples were collected, is highlighted by a red oval. **(B)** Distribution of *Nothoceros aenigmaticus* in the Southern Appalachians showing the spatially segregated sexes. Most watersheds drain in the Tennessee River, except the Coosa River which drain into the Alabama River. The populations are labeled Pigeon River and Little Pigeon River (surrounded by a blue oval), Balsam Mountain Preserve (blue oval), Little TN River and Hiwassee River (surrounded by a reddish oval) and Ocoee River and Coosa River (surrounded by an orange oval). Black dots represent sampling sites for female plants and blue dots for male plants.

The present study focuses on the southern Appalachian (SA) hornwort *Nothoceros aenigmaticus* J. C. Villarreal and K. D. McFarland, a perennial dioicous hornwort that is locally abundant on rocks along or in streams. Schuster (1992) described this species and considered it to be endemic to the SA, i.e., North Carolina (NC), Georgia (G), and Tennessee (TN) (Figure 1). Later, the species was found in Mexico, as well as in alpine neotropical regions (Villarreal et al., 2012a, b). The original description was based entirely on female plants collected in the Little TN River, which connects to the TN River (Figure 1). Male plants were subsequently found in watersheds of the Pigeon and Little Pigeon River system (Renzaglia and McFarland, 1999) and in the Little TN by Ken McFarland at Balsam Mt (TENN Herbarium). The latter being ca. 48 km upstream from the nearest female population in that same watershed. In Mexico, the species occurs in two populations where male and female plants grow together (Villarreal et al., 2012a, b).

The isolation of sexes in SA may account for the lack of known sexual reproduction, which would further be compromised by the premature abortion of motile male gametes (Renzaglia and McFarland, 1999). In addition, complete maturation of hornwort antheridia requires a short desiccation, which is absent in the *quasi* permanent aquatic habitat of *N. aenigmaticus*. These factors might contribute to the males’ inability to develop and release functional sperm cells (Renzaglia and McFarland, 1999). In the absence of fertilization, and thus sporophyte and subsequent spore formation, dispersal is achieved only via fragmentation of the gametophyte margins, and hence reproduction is purely clonal. Headwater springs of most of the streams investigated had large colonies of *N. aenigmaticus* present. Colonies were observed long distances down these streams, thus downstream dispersal is likely the primary direction for dispersing vegetative propagules. Based on that, we used a phylogenetic and population genomic approach (i) to estimate the divergence time of the split between US and Mexican populations, (ii) to verify that US populations of *N. aenigmaticus* are strictly clonal and (iii) to assess and confirm the extreme sex ratio and clonal structure of the species in the SA.

We used a published dataset (Villarreal and Renner, 2014) to date the timeframe of the SA origin of *N. aenigmaticus.* Divergence time between Mexican and US populations will show when the apparent geographic isolation of the female and male gametophytes in the US started and potentially explain the causes of the extreme sex allopatry in Appalachian plants. We analyzed 10 microsatellite loci distributed across all genomic compartments (plastid, mitochondrion and nucleus) for 273 collections of the species from seven populations in the SA and two in Mexico. Additionally, we selected 86 individuals (80 from the US and six from Mexico) and used genotyping by sequencing (GBS) methods to sample SNP markers to document species’ genetic diversity and contrast it to the estimates based on microsatellites.

## Materials and Methods

### Dating Analyses

We used a dataset derived from Villarreal et al. (2012a) and Villarreal and Renner (2014) to provide a time frame for the divergence of the US and Mexican *N. aenigmaticus*. Dating relied on a Bayesian divergence time estimation as implemented in BEAST 1.8.3 (Drummond et al., 2012). Our inferences are based on all markers for a total of 3,398 aligned nucleotides of four plastid loci (*rbc*L, *trn*L-F intron, *rps*4-trnS spacer and *mat*K). We restricted the sampling to the genus *Nothoceros* (R. M. Schust.) J. Haseg, and three outgroup genera (*Dendroceros* Ness, *Megaceros* Campb. and *Phaeomegaceros* R. J. Duff, J. C. Villarreal, Cargill & Renzaglia) based on Villarreal et al. (2015). The ingroup included 23 accessions belonging to American species of *Nothoceros* (Supplementary Table S1), such as *N. canaliculatus* J. C. Villarreal, Hassel & N. Salazar (1), *N. endiviifolius* (Mont.) J. Haseg. (2), *N. fuegiensis* (Steph.) J. C. Villarreal (2), *N. minarum* (Nees.) J. C. Villarreal (2), *N. renzagliensis* J. C. Villarreal, L. V. Campos & Uribe (1), *N. schizophyllus* (2), *N. superbus* (1), *N. vincentianus* (4) and, *N. aenigmaticus* (7). For the latter, we sampled accessions from high tropical elevations (>3,000 m, Paramos) as well as samples from US and Mexico. One individual of *N. giganteus* (Lehm. & Lindenb.) J. Haseg. from New Zealand was also included. We used a single calibration on the most recent common ancestor (MRCA) of the genus *Nothoceros*. We implemented a uniform distribution with a minimum age of 16 Mya and a maximum age of 42 Mya, following the dates estimated by Villarreal et al. (2015) for the crown age of *Nothoceros.*

Two tree priors were used (birth-death and Yule priors). Runs were performed using a relaxed clock model; the latter with uncorrelated and log-normally distributed rate variation across branches. The Markov Chain Monte Carlo (MCMC) was run for 30 million generations using the relaxed clock model, with parameters sampled every 1,000 generations. Tracer v1.5 (Rambaut and Drummond, 2007) was used to assess effective sample sizes for all estimated parameters (over 200) and to judge the percentage of burn-in for tree constructions. Trees were combined in TreeAnnotator 1.6.1 (Rambaut and Drummond, 2010), and maximum clade credibility trees with mean node heights were visualized using FigTree 1.3.1 (Rambaut, 2015). The high posterior densities (HPDs) or the smaller interval that contains 95% of the sampled values were reported. We performed a maximum likelihood (ML) analysis on RAxML (Stamatakis, 2014) using the Cipres Science Gateway.^1^ The substitution model GTR+ CAT was used with the unlinked partitions and the statistical support was evaluated using 500 ML bootstrap replicates using the same substitution model.

### Identification of Populations and Sampling Effort

We defined nine populations, seven from the SA: Pigeon River, Little Pigeon River, Balsam Mountain Preserve, Little TN River, Hiwassee River, Ocoee River and Coosa River; and two from Mexico: Zempoala and Diamantes (Figure 1 and Table 1). Our sampling of *N. aenigmaticus* comprised 273 individuals, of which 250 were from the SA and 23 from two localities in Mexico (Table 1). In the SA, the species is present in two main rivers, the TN River and the Coosa River (Figure 1A). The former is one of the largest tributaries of the Ohio River and drains into the Mississippi river, while the Coosa River drains into the Alabama River (Gulf of Mexico) (Figure 1A). We collected in five watersheds of the Tennessee River (Pigeon River, Little Pigeon River, Little TN River, Hiwassee River and Ocoee River Ocoee), and one watershed of the Alabama River (Coosa River). Some male populations from the Little TN River were collected in Balsam Mountain Preserve, ca. 48 km upstream from the female populations of this watershed (Figure 1B).

**TABLE 1:** Main characteristics of the populations of *Nothoceros aenigmaticus* sampled in this study, including watersheds, main rivers, sex and number of individuals sampled for microsatellites and GBS analysis.

Population name (watershed)	Main river/country	Sex	No. microsatellites	No. GBS
Pigeon River	Mississippi River (United States)	Males	44	18
Little Pigeon River	Mississippi River (United States)	Males	61	23
Balsam Mountain Preserve	Mississippi River (United States)	Males	10	0
Little TN River	Mississippi River (United States)	Females	62	17
Hiwassee River	Mississippi River (United States)	Females	37	14
Ocoee River	Mississippi River (United States)	Females	28	2
Coosa River	Alabama River (United States)	Females	8	6
Zempoala	Lagunas de Zempoala (Mexico)	Females/males	18	6
Los Diamantes	Cascada de los Diamantes (Mexico)	Females	5	0

**FIGURE 2:**
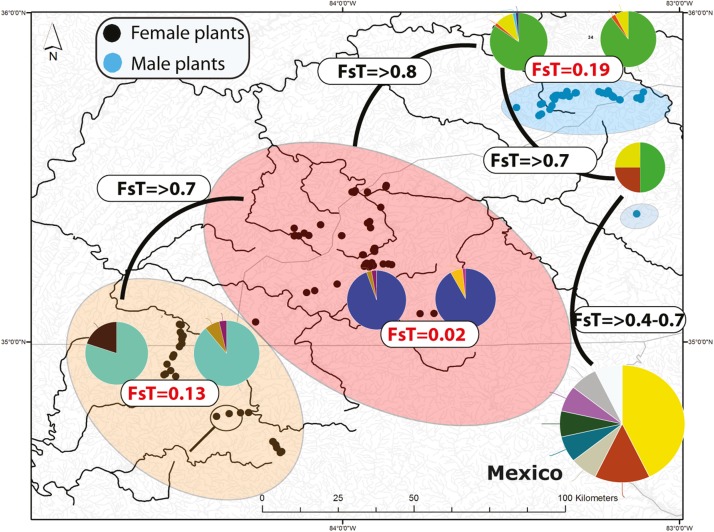
Distribution of microsatellite multilocus nuclear genotypes (MLG) and spatially segregate sexes of *Nothoceros aenigmaticus* in the Southern Appalachians. The pies represent the proportion of nuclear MLGs. Watersheds that share at least one MLG are color-coded. The Pigeon and Little Pigeon Rivers (blue oval); Balsam Mountain Preserve (oval); Little TN and Hiwassee Rivers (reddish oval) and Ocoee and Coosa Rivers (orange oval). The two localities draining into the Coosa River are circled within the Ocoee/Coosa River cluster (orange oval). Black dots represent sampling sites for female plants and blue dots for male plants. Fst values from the analyses of all microsatellites (organellar and nuclear) show low Fst values amongst contiguous watersheds (Table 2).

All collections in a stream were made from colonies at least 2 m apart. Of the 250 collections, less than 10% were sterile (without sex organs), and these were predominantly aquatic forms. In these cases, samples were cultured in the lab to verify the presence of sex organs. *Nothoceros aenigmaticus* produces abundant archegonia (Schuster, 1992, his Figure 2) and the identification is straightforward. Sex verification for putative male aquatic plants were not possible. Male plants were found exclusively in Pigeon River, Little Pigeon River and Balsam Mountain Preserve. In the remaining watersheds, only female plants were observed (Figure 1 and Table 1).

In Mexico, *N. aenigmaticus* had rarely been collected and seems restricted to high elevation (above 3,000 m) in the Neovolcanic Belt (Villarreal et al., 2012a, b). Despite an intensive search, the species was found just in two localities. The first locality was a dormant Pleistocene volcano in Las Lagunas de Zempoala, state of Morelos, where five different streams were sampled. The second locality was in Cascada de Los Diamantes (hereafter Diamantes), State of Mexico. In the former case, males and females inhabit the same patch and produced sporophytes; whereas in Los Diamantes the patches were exclusively composed of female plants.

### Population Genetic Based on Microsatellite Data

#### Microsatellite Preparation and Sequencing

Three mitochondrial (mit1, mit3, mit4), three plastid (cp44, cp45, cp59) and four nuclear (nuc35, nuc38, nuc42, nuc53) microsatellite loci were selected for use in the population study (Villarreal et al., 2012b). All samples were genotyped by multiplexing and scored using GeneMarker version 1.5 (Soft Genetics, State College, PA, United States). The marker nuc35 had a hexa-nucleotide repeat (GAGCTT). Fragment analyses revealed a single peak but inconsistent changes in repeat number. In order to verify the nature of the repeats in the locus nuc35, we used Sanger sequencing of this microsatellite for all individuals and found indels (3 or 4 bp) in some of the accessions. Manual alignment was carried out using Se-Al version 2.0a11 (Rambaut, 2002); because of low quality at the beginning and ends of the reads the matrix was trimmed to an aligned length of 171 bp. Cross-validation of exemplars from most populations was done with fragment analyses to confirm the presence of a single peak for the locus.

#### Analyzing the Chlorotype, Mitotype, and Nuclear Microsatellites

Because of the non-recombining nature of the plastid and mitochondrial genomes, each allelic combination at the three plastids (hereafter chlorotypes) and two mitochondrial (mitotypes) loci were treated as an allele at a single locus. The locus mit4 proved to be difficult to score in the Mexico and United States (US) individuals and was excluded from downstream analyses.

For the chlorotype and mitotype microsatellites, haplotype diversity was estimated as the number of haplotypes per geographical region divided by the total number of samples (P). In addition, the number of private haplotypes (G_*P*_); haploid genetic diversity (h) and unbiased haplotype diversity (uh) were calculated.

Φ_PT_ (analogous to F_st_) was estimated for chlorotypes and mitotypes (Φ_PT_ = $\frac{V\,a\,p}{\left(V\,a\,p-V\,w\,p\right)})$ where *Vap* is the variation among populations and *Vwp* is variation within populations, as suggested for haploid data, Φ_PT_. All statistical analyses were done with GenAlex 6.4b5 using 1000 permutations (Peakall and Smouse, 2006) and excluding individuals with missing data. From a total of 273 individuals and after filtering for missing-data, 267 individuals were included for the plastid analysis and 263 for mitochondrial.

For the nuclear microsatellites, 255 individuals were included after filtering for missing data; then allele frequencies, the number of alleles per locus (Na) and number of effective alleles (Ne) were calculated using GenAlex 6.4b5 (Peakall and Smouse, 2006).

#### Clonal Structure Using All Microsatellite Data

Given the clonal nature of the species, all microsatellites were analyzed together to determine the number of unique haplotypes among the clones. Clonal assignments were made using genetic distance under the stepwise model with different thresholds between individuals (Geno Dive version 2.0b2, Meirmans and Van Tienderen, 2004). If the genetic distance between a pair of individuals is below a set threshold, they will belong to the same clone. We tried several thresholds, but all resulted in a drastic reduction of the number of clones (e.g., a threshold of 0–3 gives between 74 and 70 clones, a threshold of 4–5 give 43 clones, a threshold of 6 gives 32 clones and a threshold of 9–10 gives 26 and 27 clones, respectively). One method to choose the threshold is to use the distance to the nearest neighbor. The distances between each sequence is calculated and the histogram is generated, and the threshold is choosing between the modes. In our case, the mode is between thresholds 9 and 12. However, the number of clones is drastically reduced (27 clones) using a threshold of 9. We decided to take the most conservative approach and present no threshold (0), and a threshold of 4 and the threshold of 9 for the all microsatellites.

Clonal diversity for each threshold was quantified from all microsatellites together and values of several indexes, such as Nei’s genetic diversity and evenness (eve = eff/G) were estimated. Additionally, we also calculated the ratio between genets (G) and the total number of ramets sampled (N). This ratio (G/N) is another measure of clonality where G is identified as unique multilocus genotypes by genetic markers. As G/N approaches 1/N, the signal of clonality is strong. The indexes were calculated in GenoDive version 2.0b2 (Meirmans and Van Tienderen, 2004).

Fst values were computed in GenoDive version 2.0b2 (Meirmans and Van Tienderen, 2004), and *p*-values estimated based on 1000 permutations using a stepwise mutation model. A K-Means clustering were done by calculating the allele frequencies removing the source of the individual genotypes, the K-means clustering does not assume Hardy–Weinberg equilibrium. The Bayesian information criterion and Calinksi and Harabasz pseudo –F were used to assess the optimal value of K using 50,000 simulated annealings and 20 random repeats. Additionally, an assignment analysis was conducted to assign an individual to a population based on the allele frequencies and determine potential migrants.

### Population Genomics Based on GBS Data

#### Library Preparation and Sequencing

We included 86 collections from seven populations in the GBS approach (Table 1). A double digest GBS library (enzymes Pstl/Mspl) (Abed et al., 2019) was prepared and sequenced on an Ion Proton instrument by the Plateforme d’analyses génomiques [Institut de Biologie Intégrative et des Systèmes (IBIS), Laval University, Quebec City, QC, Canada].

#### GBS Data Processing

Raw data were assessed for quality with the fastQC version 0.11.3 (Gordon and Hannon, 2010). Reads demultiplexing, filtering, and trimming were accomplished using the *process_radtags* option from the pipeline software Stacks version 2.3 (Catchen et al., 2011). To uniform the reads’ length, the sequence length distribution of fastQC was checked and reads were trimmed to 125 bp. The script was run as follow *–inline_null –renz_1 pstI –renz_2 mspI -c -r -t 125 -q -s 10*. Sequences reads were mapped to the nuclear transcriptome of *N. aenigmaticus* (SRA: ERS368224) (Wickett et al., 2014) using the software package BWA version 0.7.17 (Li and Durbin, 2009) and the SAMtools suite (Li, 2011) to convert the output of BWA to BAM format as required for Stacks (Rochette and Catchen, 2017). We created loci and identified SNPs with *gstacks* option (Stacks pipeline) on the full dataset. We concluded the SNPs calling filtering with *populations* (Stacks pipeline) applied to two different datasets, one including all GBS individuals (86) and a second one excluding those from Mexico (80 individuals). We set up the maximum accepted heterozygosity at 0 (*–max-obs-het*) because all samples were haploid gametophytes. Minimum minor allele frequency was 0.05 (*–min-maf*). The setting *populations* provides filtering options to only include loci that occur at certain frequencies in each population. We considered all individual a single population and eliminated loci that were missing from more than 50% of individuals (*–R*).

#### Phylogenetic Inference

A phylogeny of *N. aenigmaticus* was performed for both dataset (86 individuals from Mexico and US and 80 individuals from US, with known sex) including only unlinked SNPs (–*write-single-snp*). The matrices were used for a RAxML v8.2.9 (Stamatakis, 2014) maximum likelihood analysis. A bootstrap analysis with 100 replicates was run under the GTR + Γ model. The resulting phylogenetic trees were visualized and rooted using FigTree v1.4.3.

#### Genetic Diversity and Analysis of Population Structure

The genetic diversity was estimated for the SNP matrix including Mexico and US individuals (86). Statistics were calculated with the Stacks program *populations* including the *–fstats* option to have the Fst values for each pair of populations, with *p*-values describing if the Fst measures were statistically significant according to Fisher’s Exact Test. Each watershed was originally considered a different population, then individuals were split into seven groups. At least 50% of the individuals inside a population should share a locus to process it (*–r 0.5*). The maximum accepted heterozygosity was set up at 0 (*–max-obs-het*) and the minimum minor allele frequency was 0.05 (*–min-maf*).

To assess genetic structure and sex-ratio in SA, we excluded samples from Zempoala (Mexico). The SNP matrix for 80 US individuals (410 SNPs) was used hereafter. The SNP matrix was converted into a *genind* object from the R *adegenet* version 2.0.2 (Jombart and Ahmed, 2011) and used to investigate genetic structure among the individuals by a principal component analysis (PCA) using the *dudi.pca* function in the R package *ade4* version 1.7-13 (Dray and Dufour, 2007). Based on PCA results, the genetic diversity statistics were estimated. In this case, contiguous watersheds were considered a single population hence, the 80 individuals were grouped into three clusters: Pigeon and Little Pigeon Rivers (*n* = 41), Little TN and Hiwassee Rivers (*n* = 31), and Ocoee and Coosa Rivers (*n* = 8) (Figure 1B). Fst values between pairwise groups were calculated and *p*-values provided by Stacks. The same filters applied before were used (*–max-obs-het 0 –min-maf 0.05 -r 0.5 –fstats*).

We inferred population structure based on nearest neighbor haplotype co-ancestry using the fineRADstructure package (Malinsky et al., 2018), which is specially designed for RADseq data. Following the manual, we first converted the haplotypes output from *populations* (Stacks pipeline) to a *fineRADpainter* input file, reducing the maximum number of SNPs allowed at a locus to 10. We re-ordered the loci with the script provided (*sampleLD.R*), as suggested by authors. Afterward, the co-ancestry matrix (*RADpainter*) was calculated, the individuals were assigned to populations and the coalescence tree was built (*finestructure*). The outputs were loaded into the program Finestructure GUI for visualization.

#### Clonal Structure Using SNPs Data

We used a similar approach to that used for microsatellites to assess clonal diversity on GenoDive version 2.0b2 (Meirmans and Van Tienderen, 2004). We used the dataset including Mexico and US individuals (86) and set a threshold of 0 using the infinite allele model and using the setting “*clones specific to each population.*” Higher thresholds were evaluated but all resulted in a drastic reduction of the number of clones. Clonal diversity was quantified from the multilocus nuclear genotypes and values of several indexes, such as Nei’s genetic diversity and evenness (eve) were estimated. Additionally, we also calculated the ratio between genets (G) and the total number of ramets sampled (N). The indexes were calculated in GenoDive version 2.0b2 (Meirmans and Van Tienderen, 2004).

Pairwise Fst values were also computed in GenoDive version 2.0b2 (Meirmans and Van Tienderen, 2004), and *p*-values estimated based on 1,000 permutations. A K-Means clustering were done by calculating the allele frequencies removing the source of the individual genotypes. The Bayesian information criterion and Calinksi and Harabasz pseudo –F were used to assess the optimal value of K using 50,000 simulated annealings and 20 random repeats. Additionally, an assignment analysis was conducted to assign an individual to a population based on the allele frequencies and determine potential migrants.

### Spatial Genetic Structure (SGS) Using All Microsatellite Data

Based on the genetic groups derived from the phylogeny and the PCA analysis, we conducted a SGS test using microsatellites. Spatial autocorrelation analysis of kinship among ramets was carried out using SPAGeDi 1.5. (Hardy and Vekemans, 2002). Contiguous watersheds were examined at the ramet level (e.g., Pigeon and Little Pigeon Rivers; Little TN and Hiwassee Rivers; and Ocoee and Coosa Rivers). The average multilocus kinship coefficient (F_ij_) was computed according to Loiselle et al. (1995) and averaged within each of the chosen eight distance classes: 0.25, 1, 5, 10, 20, 30, 40, 50 km. Average values of F_ij_ were regressed on the natural logarithm of distance ln(*d*_ij_) in order to obtain the regression slope, *b*, which quantifies the SGS. To test for SGS under the null hypothesis of no correlation of F_ij_ and ln(*d*_ij_), the spatial positions of individuals were permuted 10,000 times.

## Results

### Dating Analyses

The divergence times for the two nodes of interest are shown in Supplementary Figure S1 and Table S2. The MRCA to the crown group from Neotropics, Mexican and US *N. aenigmaticus* populations dates to **3.89** (1.03–7.76) Mya using a birth-death prior or **4.35** (1.16–8.4) Mya with the Yule prior. The diversification of the US/Mexican *N. aenigmaticus* crown group dates to **0.79** (0.061–1.93) Mya using a birth-death tree prior, or **0.88** (0.06–2.13) Mya with a Yule prior (Supplementary Figure S1 and Table S2).

### Population Genetic Based on Microsatellites Data

#### Genetic Diversity

The percentage of missing data for chlorotypes and mitotypes was 2.1 and 3.6%, respectively. No chlorotype or mitotype was shared between male and female populations. The highest chlorotype diversity occurred in Zempoala, Mexico (*h* = 0.796; males and females), followed by SA populations in the Little Pigeon (*h* = 0.496), Pigeon (*h* = 0.328), and Little TN (*h* = 0.211) Rivers (Supplementary Table S3). The number of mitotypes (i.e., 39) was always higher than the number of chlorotypes (i.e., 24; Supplementary Table S3 and Figure S2). Zempoala, Mexico harbored the highest diversity of mitotypes (*h* = 0.86), followed by the Little TN (*n* = 0.589), Little Pigeon (*h* = 0.517) and Ocoee (*h* = 0.502) rivers (Supplementary Table S3). Eleven chlorotypes and 16 mitotypes were found among all male populations (Pigeon, Little Pigeon and Balsam Mt.) and 10 chlorotypes and 19 mitotypes among female populations (Little TN, Hiwassee, Ocoee, and Coosa Rivers). Likewise, most mitotypes and chlorotypes were restricted to single or contiguous watersheds with limited downstream dispersal. Examples of share widespread chlorotypes are found in Little TN and Hiwassee River (Supplementary Figure S2). Similarly, one chlorotype is shared between the Coosa River and Ocoee River and one is restricted to the headwaters of the Ocoee River (Supplementary Figure S2). Regarding nuclear diversity, 20 microsatellite alleles are distributed across the four microsatellite loci (Supplementary Table S4 and Figure 2). The missing data composed less than 2% of the total data. The number of alleles per marker ranged between 2 and 10. The most variable marker (nuc35) has 10 alleles (Supplementary Table S4).

#### Population Differentiation

Average Φ_ST_ values for chlorotypes are high (0.592; p. 0.01) with low levels of pairwise Φ_PT_ between Little TN and Hiwassee Rivers (0.00) and between Zempoala and Cascada de Los Diamantes (0.099) and moderate values between Pigeon and Little Pigeon Rivers (0.272). Average Φ_PT_ values for mitotypes are high (0.409; p. 0.01) with low levels of pairwise Φ_PT_ between Little TN and Hiwassee Rivers (0.013), between Zempoala and Cascada de Los Diamantes (0.035) and between Pigeon and Little Pigeon Rivers (0.004) (Supplementary Table S5). In general, Φ_PT_ values are low between contiguous watersheds and the differentiation between non-contiguous watersheds is high (Supplementary Table S5).

#### Clonal Structure Using All Microsatellites

Seventy-two, 43 and 26 clones (thresholds 0, 4, and 9) were detected using GenoDive (Supplementary Table S6). With no threshold, the number of clones is high in Little TN River (13), Ocoee River (11), Pigeon River (15) and Little Pigeon River (24). With a threshold of 4, the number of clones in Little TN River was drastically lower (five clones). There is evidence of migrants between contiguous watersheds and two cases of potential migrants between non-contiguous watersheds. One migrant shared by Little TN and Pigeon River and a second one shared by Zempoala and Ocoee Rivers. No multilocus genotype was shared between sexes. The Fst values for nuclear data show low values between Pigeon/Little Pigeon River (0.191), Little TN/Hiwassee Rivers (0.026) and Ocoee/Coosa Rivers (0.133). The Fst values are much higher between non-contiguous watersheds (Table 2) and a Fst value of 0.556 between Balsam Mountain Preserve and Mexican populations (Table 2).

**TABLE 2:** Pairwise Fst values among populations of *Nothoceros aenigmaticus*.

	Little TN River	Hiwassee River	Ocoee River	Coosa River	Pigeon River	Little Pigeon River	Balsam Mt. Preserve	Zempoala Mexico	Diamantes Mexico
Little TN River	–	0.152	0.988	0.981	0.981	0.972	–	0.936	–
Hiwassee River	0.026	–	0.9859	0.987	0.980	0.971	–	0.933	–
Ocoee River	0.787	0.819	–	0.057	0.980	0.966	–	0.907	–
Coosa River	0.827	0.883	0.133	–	0.980	0.964	–	0.922	–
Pigeon River	0.828	0.857	0.750	0.785	–	0.120	–	0.932	–
Little Pigeon River	0.803	0.821	0.717	0.762	0.191	–	–	0.919	–
Balsam Mt. Preserve	0.802	0.871	0.697	0.827	0.775	0.725	–	–	–
Zempoala Mexico	0.704	0.743	0.534	0.558	0.589	0.566	0.440	–	–
Diamantes Mexico	0.809	0.873	0.737	0.843	0.760	0.715	0.753	0.356	–

*Values below the diagonal for all microsatellites and above the diagonal for SNPs (GBS). The values from microsatellite data were significant after 1000 permutations at a *p*-value of 0.01.*

The K-Means clustering recovered six groups (*k* = 7), namely Little Pigeon, Pigeon Balsam Mountain Preserve, Little TN/Hiwassee, Ocoee/Coosa, and Zempoala/Diamantes (Supplementary Table S7). Population assignment analyses recovered migrants between contiguous watersheds, bus also two potential migrants between Little TN and Little Pigeon River and between Coosa River and Zempoala (Mexico).

### Population Genetic Based on GBS Data

#### GBS Inferences

Approximately 88 million sequencing reads were generated for the 86 samples. The total number of reads retained after demultiplexing, filtering and trimming was 64,836,726, varying between 18,885 for sample MA42 to 4,029,405 for sample MA237. Retaining only reads that mapped to the transcriptome reference, reduced the number of reads drastically, e.g., to 2,621 for MA42 and from 4,021,346 to 109,298 for the best mapped sample (MA135). An average of 43% of the reads mapped to the reference transcriptome was retained for *gstacks* (Stacks pipeline) to create 6,157 loci (at least 5X mean coverage), of which 99% had BLAST hits on the transcriptome of *N. aenigmaticus*. Table 3A summarizes the number of loci and SNPs for each dataset (including and excluding Mexican samples) before and after filtering with the Stacks pipeline (maximum accepted heterozygosity of 0, SNPs with minor allele frequency greater than 0.05 and more than 50% coverage).

**TABLE 3A:** Number of loci and SNPs of *Nothoceros aenigmaticus* for both dataset (exclusively United States and US plus Mexico), before and after filtering with the option populations of the Stacks pipeline *(–max-het-obs 0 –min-maf 0.05 –R 0.5*).

Matrices	Prefilter		Post-filter		
		
	No. loci	No. SNPs	No. loci	No. SNPs	No. unlinked SNPs
Mexico and US individuals	6,157	1,399	2,402	669	491
US individuals	6,157	823	2,368	410	305

#### Phylogenetic Inference

**FIGURE 3:**
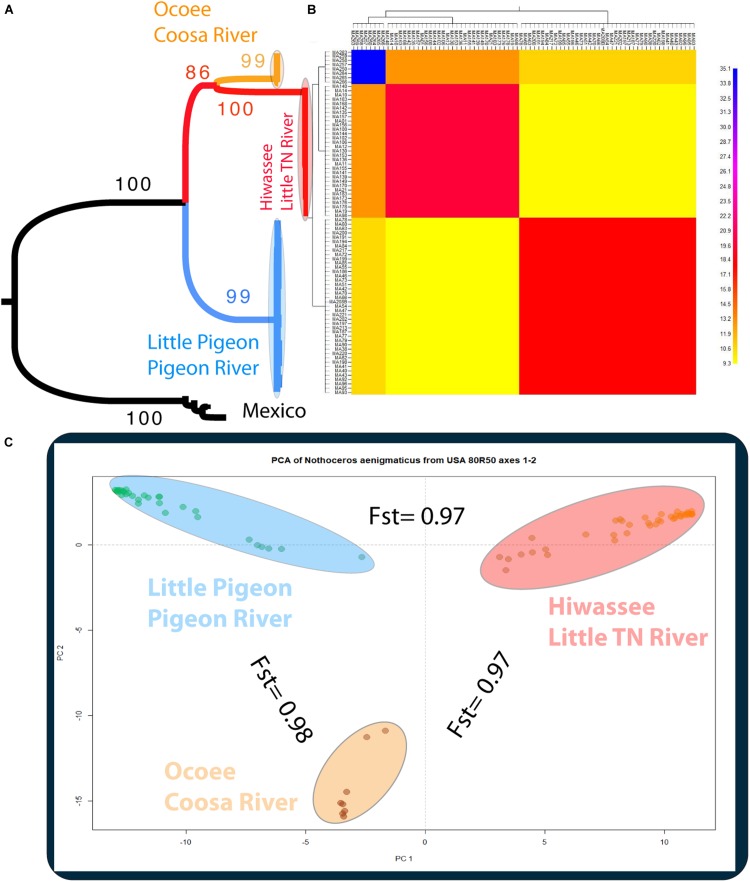
**(A)** Phylogenetic tree inferred from the Mexican and United States GBS data of *Nothoceros aenigmaticus* (86 samples SNPs matrix). The clade of each genetic group is highlighted. Bootstrap values are indicated at the branches. **(B)** Clustered fineRADstructure co-ancestry matrix of *N. aenigmaticus* from the United States estimated from the unlinked SNPs matrix (80 samples). The heatmap shows pairwise co-ancestry between individuals, with blue representing the highest level, red and orange indicating intermediate levels, and yellow representing the lowest levels of shared co-ancestry. **(C)** Principal Components Analysis (PCA) of 80 samples of *N. aenigmaticus* from the United States (SNPs matrix) indicating Fst values between groups. Colors correspond to those in Figure 1.

The phylogenetic relationships of the 86 samples (Mexico and US individual) were inferred from 491 SNPs (Table 3A) with 20.4% of missing-data. The samples are clustered in two highly supported clades (MLBS = 100) corresponding to the Mexican and US individuals of *N. aenigmaticus* (Figure 3A). The US samples were further resolved in three well-supported clades (MLBS > 85) (Figure 3A): the first includes the male plants from Pigeon and Little Pigeon Rivers, the second one consists of female plants from Little TN and Hiwassee, and the third one contains specimens from Ocoee plus Coosa Rivers (females). Within each clade, the relationships are ambiguous. The matrix of the 80 US samples had 305 SNPs (Table 3A) and 19.9% of missing-data, and its analysis yielded the same topology and it is not shown here.

#### Genetic Diversity and Population Structure

Using the SNP matrix containing 86 individuals, we obtained a total of 6,157 loci. Among them, 2,930 were present in at least 50% of the individuals inside a population (watershed) (filter −*r 0.5*). Broadly, levels of nucleotide diversity (π) were low, ranging from 0 (Ocoee River) to 0,05436 (Zempoala) (Supplementary Table S8). The number of polymorphic sites inside a population varied from 0 (Ocoee River) to 10 in US watersheds (Supplementary Table S8). In Zempoala (Mexico), a population with sympatric sexes, this number increased to 93. Private sites were also particularly abundant among Mexican plants with 307 unique alleles, and comparatively really low within the US populations with values between two to eight (Supplementary Table S8). Pairwise Fst values showed little differentiation between contiguous rivers (watersheds), in contrast to the high differentiation between non-contiguous rivers (Table 2).

The PCA plot (Figure 3C) based on the US matrix 410 SNPs from 80 samples reflected the most likely phylogenetic relationships (Figure 3A). Individuals of *N. aenigmaticus* from the US clustered in three main groups reflecting the contiguity of watersheds: one comprising samples from Pigeon and Little Pigeon Rivers (males), the second one samples from Little TN-Hiwassee Rivers (females) and the third samples from Ocoee/Coosa Rivers (females). Pairwise Fst values among these three groups were high and agreed with phylogenetic and PCA data. Fst value between Little TN-Hiwassee and Ocoee/Coosa Rivers was 0.98 with *p*-values < 0.005 for 234 of the 239 scaffolds compared; Fst value for Little TN-Hiwassee and Pigeon-Little Pigeon Rivers was 0.97 with *p*-values < 0.005 for 299 of the 304 scaffolds compared; and the Fst between Ocoee/Coosa and Pigeon-Little Rivers was 0.97 with *p*-values < 0.005 for 266 of the 274 scaffolds compared. The Fst value between sexes was also high (0.65) (*p*-values < 0.005 for all the scaffolds compared). In terms of genetic diversity considering three populations (contiguous watersheds), 2,571 loci (of 6,157) were shared by at least 50% of the individuals within each population. The loci were composed of 320,884 sites; 412 variants. Levels of nucleotide diversity (π) were low (<0.006 for variant positions and < 0.00001 for variant and fixed positions) (Table 3B). Following the same trend, variation within each population never exceeded 10 polymorphic sites. However, the number of private or unique alleles within a population highly increased when individuals were grouped by contiguous watersheds (Table 3B). In this case, it ranged from 99 to 168, compared to two and eight in Supplementary Table S8 (individuals grouped by watersheds).

**TABLE 3B:** Population statistics for polymorphic loci and for both polymorphic and fixed loci. Filters applied were *–max-het-obs 0 –min-maf 0.05 –r 0.5*.

Watersheds	Variant positions			Variant and fixed positions						
		
	*N*	Private	π	*N*	Private	Sites	Variant sites	Polymorphic sites	PPL %	π
Pigeon and Little Pigeon Rivers	26	128	0.0035	26	128	309,062	409	7	0.0023	0.00000
Little TN and Hiwassee Rivers	32	168	0.0061	32	168	298,544	405	10	0.0034	0.00001
Ocoee and Coosa Rivers	7	99	0.0006	7	99	250,941	409	1	0.0004	0.00000

*Statistics include the total average number of individuals genotyped at each locus (N), the number of alleles unique to each population (private), mean nucleotide diversity (π), the total number of sites for each populations (sites), the number of sites across all populations that are variant (variant sites), the number of sites inside each population that are variant (polymorphic sites) and the percentage of polymorphic sites (PPL %).*

According to the fineRADstructure results (Figure 3B), the three geographic clusters shared more co-ancestry within each other than between them. Comparison between clusters shows that individuals of *N. aenigmaticus* from Ocoee plus Coosa Rivers (females) share more co-ancestry with individuals from Little TN plus Hiwassee Rivers (females) than with those from Pigeon and Little Pigeon Rivers (males) (Figure 3B).

#### Clonal Structure

The total number of clones (Num) was 31 (when the clones were made specific to each of population) or nine (clones not specific to each population). A single clone occurs in each US river, except Hiwassee and Coosa River (two clones each), which suggests prominent clonality in the species in SA. In Mexico, the population of Zempoala had five clones. The G/N ratio was low (G/N < 0.1) in Pigeon, Little Pigeon, Little TN and Hiwassee Rivers. The collections from Ocoee and Coosa Rivers show higher G/N ratio (i.e., 0.5 and 0.33, respectively; Supplementary Table S9), and Zempoala presents the highest ratio (G/N = 0.83). Pairwise Fst values were lower between contiguous watersheds. For example, 0.363 between Little TN and Hiwassee River (Supplementary Table S10) and over 0.9 between the non-contiguous watersheds (Supplementary Table S10). Population assignment recovered migrants between contiguous watersheds, especially between Little TN and Hiwassee and Pigeon/Little Pigeon River (Supplementary Tables S11, S12). The K-Means clustering recovered five groups (*k* = 5), namely Little TN/Hiwassee Rivers, Ocoee River, Coosa River, Little Pigeon/Pigeon Rivers and Zempoala (Supplementary Tables S11, S12).

#### Spatial Genetic Structure Using All Microsatellite Data

At the ramet level, the regression of F_ij_ over the natural logarithm of the geographic distance produced a negative slope for the three contiguous watersheds (Supplementary Table S13). A significant average F_ij_ was estimated up to 10 km in Pigeon and Little Pigeon Rivers; in 1 km in Little TN and Hiwassee Rivers, whereas in Ocoee and Coosa Rivers, it was significant in 0.25 km and further in third, fourth and fifth distance classes (5, 10, and 20 km, respectively) (Supplementary Figure S3). At the genet level, the kinship-distance regression slopes were also negative for the three contiguous watersheds (Supplementary Table S13). The average significant values of F_ij_ in genets were found in the two first distance classes (up to 1 km) in Pigeon and Little Pigeon Rivers; in km 1 and 10 in Little TN and Hiwassee Rivers; while in Ocoee and Coosa Rivers, it was significant in the third (5 km) and the fifth class (20 km) (Supplementary Figure S3).

## Discussion

The evolution of separate sexes (dioicy) has intrigued biologists for centuries. The genetic advantages of separate sexes are evident (e.g., avoid inbreeding, providing opportunities for outcrossing and recombination enhancing haplotype diversity), however, the costs of dioicy outnumber the theoretical benefits (e.g., Obeso, 2002). This is particularly evident in plants with spatially separated sexes, which in the most extreme cases may lead to a lack of sexual reproduction. In this study, we analyzed the population structure of a haploid plant with extreme sex ratio (either male or female populations), degenerate male gametes and extended clonality. We here show and confirm (1) the divergence times between Mexican and US plants dating back to the Pleistocene, (2) the strictly clonal nature of the US populations and (3) US populations consisting of few clones with extreme sex ratios (0:1) and high genetic structure (Fst values over 0.9 using nuclear markers). The spatial segregation of sexes within the SA is strongly supported by SNP markers and microsatellite data with shared genotypes mostly between contiguous watersheds. The SA drainage system is thought to have been remodeled by geomorphological processes during the Pleistocene, which could have led to a mixing or isolation of alleles in aquatic and rheophilous taxa (Ross, 1971; Mayden, 1988; Bossu et al., 2013) in contiguous watersheds.

### Dispersal Origin of US *N. aenigmaticus* and Extreme Sex Ratios

The crown group of alpine neotropical *N. aenigmaticus* dates to 3–4 Mya (Supplementary Figure S1 and Table S2). SA populations of *N. aenigmaticus*, a taxon formerly considered endemic to this region, share a MRCA with Mexican plants, no later than ca. 600–800,000 years (3,200 years –1.7 Mya), suggesting that the US plants experienced the last Ice age. The high differentiation between Mexican (sexual) and US (clonal) plants points to a long and on-going isolation of US populations with highly restricted contemporary migration between these two regions.

The Mexican/SA populations were probably founded following dispersal from sexually reproducing, and hence spore producing, alpine tropical populations. Although the spores do not exhibit the syndrome necessary for effective wind-mediated long distance dispersal (e.g., a thick wall; van Zanten and Gradstein, 1988), they could have been transported by lower altitude winds or even by a biotic vector, such as migratory birds (Lewis et al., 2014) as has been invoked for leafy liverworts that exhibit disjunct distributions and have similar spores with large plastids (“green-looking”) with a rather thin exine (van Zanten and Gradstein, 1988). *Nothoceros aenigmaticus* is dioicous and hence the spores are “unisexual.” Several scenarios are possible. (1) The occurrence of both sexes in the SA thus requires at least two independent dispersal events: one for male plants (Pigeon/Little Pigeon River) and the other for female plants (Little TN/Hiwassee River, Ocoee/Coosa River) and a third potentially to Balsam Mountain Gap (male plants). (2) It is possible that the current species distribution is product of a co-dispersal of male and female spores and the species was widespread in the SA region after a dispersal from Mexico in the Plio-Pleistocene. (3) Or, local unknown disturbances fragmented and isolated the populations in the US. In this case, we envision a scenario of dispersal from Paramos to Mexico/US, following local vicariance of US watersheds from Mexico.

The spatial separation of sexes (sex ratios 0:1) shown in *N. aenigmaticus* from US suggests that SA populations survived through the Pleistocene by clonal growth. The reasons for a preferential expression, survival and establishment of either sex is not yet clear in bryophytes (Bisang and Hedenäs, 2005; Baughman et al., 2017). Male spore mortality is one of the most commonly suggested factors attributed to the striking female biased ratio in bryophytes (Baughman et al., 2017). Using microsatellite data, we found three potential migrants between the Little TN watershed (female) and Little Pigeon River (male) and one between Zempoala (Mexico) and Coosa River. We cannot dismiss whether these are true migrants or reflect homoplasy in the microsatellite data, but based on SNP data the latter explanation is more likely.

### Spatial Segregate Sexes (SSS) and Spatial Genetic Structure (SGS)

The evolution of SSS in dioicous species is intriguing and rather counter-intuitive. A reduced reproductive success seems to be compensated by niche specialization (Bierzychudek and Eckhart, 1988) or high intersexual competition (Rogers and Eppley, 2015). Niche specialization lacks experimental evidence in bryophytes (Bisang et al., 2015; Brzyski et al., 2018) or empirical support in flowering plants (Rogers and Eppley, 2015). In some cases, female plants tend to live in more benign habitats such as moisture slopes in the conifer species *Austrocedrus chilensis* (D. Don) Pic. Serm. & Bizzarri (Nuñez et al., 2008) or high phosphorous substrates in the grass *Distichlis spicata* (L.) Green (Eppley et al., 2009). In the desert moss, *Syntrichia caninervis*, however, males were never found in harsh environments (Stark et al., 2005). No studies have been attempted to measure the micro-environmental conditions or nutrient concentration of the streams where male and female *N. aenigmaticus* grow to assess niche specialization. Regarding intersexual competition, Rogers and Eppley (2015) found evidence for such competition in the grass *Distichlis spicata*, suggesting that competition rather than niche specialization is driving the SSS in the species. Equally, the males from the Pigeon and Little Pigeon rivers are separated from the closest female plants by nearly 30 km (Figures 1, 2). The male population from the Balsam Mountain Preserve occurs ca. 48 km upstream from the closest known female population (Little TN River) remains a puzzle. The strict sex allopatry across watersheds suggests intersexual competition. In addition, the lack of sexual selection, combined with exclusively clonal growth likely explains the loss of the ability to produce viable sperm cells (Renzaglia and McFarland, 1999).

Our expanded sampling confirmed the SSS, especially among distant watersheds. The observed fine-scale SGS indicate that within contiguous watersheds, alleles of *N. aenigmaticus* are genetically closer between nearby individuals than between distant individuals. Gene flow is limited in the species, and it seems to occur mostly between contiguous watersheds. Usually, SGS is the result of limited gene dispersal and can be influenced by a broad array of life-history traits including clonality and selfing. In our case, clonality is a crucial determining factor because clonality increases kinship at a small spatial scale. Therefore, clonality, SGS and SSS may have an impact on the complete absence of sexual reproduction in US *N. aenigmaticus*.

Eckert (2002) summarized the ecological implications of the loss of sex for clonal plants, invoking a “use-it-or-lose-it” hypothesis for degeneration of sexual structures in apomictic plants. Potential sterility mutations may occur in clonal plants that have completely abandoned sexual reproduction and are particularly likely in populations at the margins of their range (Eckert, 2002; Barrett, 2015) and especially in dioicous taxa (Hu et al., 2017). The “use-it-or-lose-it” certainly applies to *N. aenigmaticus* and it is more evident in male gametophytes. In Mexican *N. aenigmaticus*, sexually functional male and female plants grow intermixed. Mexican male plants are small and inconspicuous (less than 4 mm wide) with abundant (>20) antheridial chambers per gametophyte, typical of dioicous hornworts (Proskauer, 1948). By contrast, US male plants have larger thalli, exuberant growth and few antheridial chambers per thallus (up to 6), which might not open and if opened, the antheridia bear abortive sperm cells (Renzaglia and McFarland, 1999). Our observations suggest that male plants are no longer constrained by sexual selection; therefore, they do not have the selective pressure to produce viable sperm cells. We could not determine whether archegonia (in female plants) degenerate and we do not know of a single study capable of assessing a degeneration of the female function in sporic plants. A detailed developmental analysis using expression data is needed to test whether the male and female function are under relaxed selection.

Estimates of divergence times suggests that clones have persisted since the Pleistocene (Supplementary Figure S1) and hence that their higher levels of genotypic diversity in plastid and mitochondrial loci, high number of nuclear private alleles (e.g., 168 for Little TN together with Hiwassee river, Table 3) may result from a high number of somatic mutations (Schuster, 1992). In contrast to many organisms in the SA (Soltis et al., 2006), a lack of northward expansion of *N. aenigmaticus* may be due for the limited dispersal capabilities (lack of spores) and lack of suitable habitats.

### Patterns of Clonal Diversity

Population genetic theory predicts that in asexual taxa, clonal growth may amplify the effect of genetic drift by reducing the effective size of local populations (Chung and Kang, 1996; Jones and Gliddon, 1999) and that genetic polymorphism will decrease over time. Using microsatellite and SNP markers, the best-sampled SA watersheds (Little TN River and Little Pigeon River) have few multilocus genotypes and little diversity (Figure 2 and Supplementary Tables S3, S4, S6), consistent with clonal growth. The high Φ_PT_ and Fst values (Table 2 and Supplementary Tables S5, S10) between non-contiguous populations are consistent with little genotype exchange between them.

The moss *Pleurochaete squarrosa* (Brid.) Lindb. has genetically highly structured populations (Grundmann et al., 2008). Its central European populations are mostly clonal, have a low G/N ratio and are well-differentiated (i.e., Gst values of 0.89) from putatively sexually reproducing Mediterranean populations. The low levels of genotypic diversity in glaciated areas seem to be a widespread pattern across European bryophytes (Kyrkjeeide et al., 2014). In lacustrine bryophytes, flow among populations is moderate (Fst values from 0.14 to 0.280), such that the genetic structure among these populations is likely best explained by clonal growth and episodic spore and diaspore dispersal (Korpelainen et al., 2012). The low values of genotypic diversity and high levels of clonal differentiation among bryophyte populations are in line with studies from dioecious island-inhabiting angiosperms with clonal propagation (Meloni et al., 2013). In bryophytes, high population differentiation (Fst values over 0.9) is rare and typically linked to transoceanic distribution (Patiño and Vanderpoorten, 2018).

Genetic diversity in aquatic plants is expected to increase downstream by increasing gene flow between isolated populations (Nilsson et al., 2010). In addition to the migration bias that would result in higher genetic diversity among downstream populations, the upstream populations may further see their genotypic diversity decline due to downstream drift (i.e., the “drift paradox”; Müller, 1974), unless the regular loss of individuals drifting downstream and resulting in the extinction of genotypes is compensated by some other means of upstream dispersal (Pollux et al., 2007; Nilsson et al., 2010). Most populations of *N. aenigmaticus* are found in the headwaters or medium-size streams, rarely in high volume rivers, suggesting that stranding and successful establishment downstream is rare. One prime example is the lack of male plants downstream in the Little TN River (just ca. 48 km upstream of the first colony of female populations) suggesting a highly limited dispersal capability of *N. aenigmaticus*. Almost every single river is separated by a mountain divide, suggesting that dispersal may be highly limited without having spores. Intercatchment dispersal mainly via water birds has repeatedly been reported in plants, particularly angiosperms (Pollux et al., 2007; Smulders et al., 2008). In the aquatic and sexually reproducing moss *Platyhypnidium riparioides* (Hedw.) Dixon, episodic dispersal of vegetative diaspores and occasional from spores contribute to the structured population dynamic of the species with very little downstream dispersal (Hutsemékers et al., 2003).

One potential explanation, and extremely challenging to test at this scale, is that *N. aenigmaticus* dispersed between catchments following changes in river geomorphology and potential post-glaciation floods (Ross, 1971; Mayden, 1988; Willet et al., 2014). We postulate that in the case of lack of sexual reproduction (and therefore spores) the dispersal via fragments could be limited and only accomplished via Pleistocene river piracy. This process has been established for the Little TN River Basin (Ross, 1971; Prince et al., 2010; Willet et al., 2014), and Ocoee and Coosa Rivers, the latter rivers draining in different watersheds (Mills et al., 2005). Recent modeling studies of the Southern US river systems have confirmed the historical dynamics of the drainages due to constant erosion and drainage changes, including stream captures (Willet et al., 2014). Testing the hypothesis of river piracy in the watersheds in our study is very challenging because of the localized spatial scale of the present study.

## Conclusion

The SA *N. aenigmaticus* is a recent immigrant from sexual tropical populations with an extreme case of spatial separation of sexes (1:0 ratio). Based on microsatellite and SNP data, some genotypes are shared between contiguous watersheds, and hence the current genetic diversity of this species is not partitioned by watersheds. The data show sex-specific SGS with watersheds displaying a single sex. The presence of only clonal *N. aenigmaticus* in the SA suggests that the species is “locked” in the SA. Given the low extant genetic diversity, which can only be increased by somatic mutations in the absence of sexual reproduction, any slight alteration of the habitat may cause the local extinction of the species. *Nothoceros aenigmaticus* may surge as a model to study the impact of clonality on the deterioration and lack of sexual function (e.g., genes involved in flagellum formation), the proliferation of somatic mutations (in organellar and nuclear genomes) and mutational meltdown in haploid plants.

## Data Availability Statement

The sequence data are already published and found in Supplementary Table S1. Microsatellite raw data used for the dating and population genetic analyses are available upon request. Raw sequences GBS data are deposited in NCBI BioProject ID PRJNA562471.

## Author Contributions

MA-G performed and analyzed GBS data, performed population genomic analyses. JV conceived the study, conducted fieldwork, developed and analyzed microsatellite data, performed phylogenetic analyses. KM planned and conducted fieldwork, provided research context, natural history and geographic data on the species. BG conceived and planned the study, provided funding. All authors read and approved the manuscript.

## Conflict of Interest

The authors declare that the research was conducted in the absence of any commercial or financial relationships that could be construed as a potential conflict of interest.

## Footnotes

1 https://www.phylo.org
