# Supplementary material for: Population Genomics and Phylogeography of a Clonal Bryophyte With Spatially Separated Sexes and Extreme Sex Ratios
Source: Front Plant Sci. 2020 May 8;11:495. doi: 10.3389/fpls.2020.00495 (PMC7226906; doi:10.3389/fpls.2020.00495)
Supplement: Supplementary file 1 [file Data_Sheet_1.docx]

**Table S1.** Voucher information and GenBank accessions numbers for specimens used in this study for the phylogenetic and dating analyses of the genus *Nothoceros*. The information is provided in the following order: Country, collector, collection number, herbarium, elevation (when available) and Genbank numbers for the following genetic markers: *rbc*L, *trnL* intron and spacer, *rps*4-*trn*S spacer, *mat*K, *nad*4-*nad*5 spacer, ITS2 respectively.

| Species | Country, collector, herbarium | Elevation | *rbc*L | trnL intron | trnL spacer | *rps*4-trnS spacer | *mat*K | *nad*4-*nad*5 spacer | ITS2 |
| --- | --- | --- | --- | --- | --- | --- | --- | --- | --- |
| *Nothoceros aenigmaticus* (R.M. Schust.) J.C. Villarreal & K.D. McFarland | Colombia, Villarreal *et al.* 1082 (COL) | 3200 m. | HM056184 | HM067481 | HM067532 | HM067583 | JN559959 | HM163425 | HM163375 |
| *Nothoceros aenigmaticus* (R.M. Schust.) J.C. Villarreal & K.D. McFarland | Costa Rica, Villarreal *et al.* 885 (INB) | 3000 m. | HM056179 | HM067476 | HM067527 | HM067578 | JN559955 | HM163420 | HM163370 |
| *Nothoceros aenigmaticus* (R.M. Schust.) J.C. Villarreal & K.D. McFarland | Costa Rica, Villarreal *et al.* 892 (INB) | 3000 m. | HM056180 | HM067477 | HM067528 | HM067579 | JN559956 | HM163421 | HM163371 |
| *Nothoceros aenigmaticus* (R.M. Schust.) J.C. Villarreal & K.D. McFarland | Mexico, Villarreal 1258 (CONN) | 3000 m. | HM056186 | HM067483 | HM067534 | HM067585 |  | HM163427 | HM163377 |
| *Nothoceros aenigmaticus* (R.M. Schust.) J.C. Villarreal & K.D. McFarland | USA, Villarreal & McFarland 1092 (CONN) | 500 m. | HM056197 | HM067494 | HM067545 | HM067596 | JN559972.1 | HM163438 | HM163388 |
| *Nothoceros aenigmaticus* (R.M. Schust.) J.C. Villarreal & K.D. McFarland | Villarreal & McFarland 965 (CONN) |  | HM056193 | HM067490 | HM067541 | HM067590 |  | HM163434 | HM163384 |
| *Nothoceros aenigmaticus* (R.M. Schust.) J.C. Villarreal & K.D. McFarland | Venezuela, Villarreal 787 (CONN) | 3400-3600 m. | HM056177 | HM067474 | HM067525 | HM067576 | JN559953 | HM163418 | HM163368 |
| *Nothoceros canaliculatus* (Pagan) J.C. Villarreal et al. | Costa Rica, Lépiz s.n. | ca. 1500 m. | HM056176 | HM067473 | HM067524 | HM067575 | JN559952 | HM163417 | HM163367 |
| *Nothoceros endiviifolius* (Mont.) J. Haseg. | Chile, Duckett s.n. (CONN) | ca. 10 m. | HM056152 | HM067449 | HM067500 | HM067551 | JN559930 | HM163393 | HM163348 |
| *Nothoceros endiviifolius* (Mont.) J. Haseg. | Chile, Allen 26401 (MO) | ca. 10 m. | HM056153 | HM067450 | HM067501 | HM067552 | JN559931 | HM163394 |  |
| *Nothoceros fuegiensis* (Steph.) J.C. Villarreal | Chile, Goffinet *et al.* 9527 (CONN) | ca. 10 m. | HM056156 | HM067453 | HM067504 | HM067555 | JN559934 | HM163397 | HM163351 |
| *Nothoceros fuegiensis* (Steph.) J.C. Villarreal | Chile, Goffinet *et al.* 9533 (CONN) | ca. 10 m. | HM056161 | HM067457 | HM067508 | HM067559 | JN559939 | HM163401 |  |
| *Nothoceros giganteus* (Lehm. & Lindenb.) J. Haseg. | New Zealand, Von Konrat 27407 (FM) | s.n. | HM056154 | HM067451 | HM067502 | HM067553 | JN559932 | HM163395 | HM163349 |
| *Nothoceros minarum* (Nees.) J.C.Villarreal | Uruguay, Cargill & Prieto 2625 |  | JX872433 | KF861841 | KF861846 | KF861835 | KF612916 | KF861824 | --- |
| *Nothoceros minarum* (Nees.) J.C.Villarreal | Brasil, Santos 411 (CONN) | -- | HM056169 | HM067466 | HM067517 | HM067568 | JN559946 | HM163410 |  |
| *Nothoceros renzagliensis* J.C.Villarreal et al. | Colombia, Villarreal *et al.* 1080 (COL) | 2968 m. | HM056162 | HM067459 | HM067510 | HM067561 | JN559940 | HM163403 | HM163357 |
| *Nothoceros schizophyllus* (Gottsche ex Steph.) J.C. Villarreal | Costa Rica, Villarreal *et al.* 895 (INB) | 700 m. | HM056175 | HM067472 | HM067523 | HM067574 | JN559951 | HM163416 |  |
| *Nothoceros schizophyllus* (Gottsche ex Steph.) J.C. Villarreal | Panama, Villarreal & Varela 584 (PMA) | 800 m. | HM056173 | HM067470 | HM067521 | HM067572 | JN559949 | HM163414 |  |
| *Nothoceros superbus* J.C.Villarreal et al. | Costa Rica, Salazar *et al.* 20676 (PMA) | 1615 m. | HM056172 | HM067469 | HM067520 | HM067571 | KF482317 | HM163413 | HM163364 |
| *Nothoceros vincentianus* (Lehm. & Lindenb.) J.C.Villarreal | Saba (Caribbean), Buck 51344 (NY) | -- | HM056170 | HM067467 | HM067518 | HM067569 | JN559947 | HM163411 | -------- |
| *Nothoceros vincentianus* (Lehm. & Lindenb.) J.C.Villarreal | Panama, Villarreal & Rodríguez 802 (CONN) | ca. 800 m. | HM056167 | HM067464 | HM067515 | HM067566 | JN559944 | HM163408 | HM163361 |
| *Nothoceros vincentianus* (Lehm. & Lindenb.) J.C.Villarreal | Venezuela, Villarreal *et al.* 769 (CONN) | ca. 2100 m. | HM056165 | HM067462 | HM067513 | HM067564 | JN559942 | HM163406 | HM163359 |
| *Nothoceros vincentianus* (Lehm. & Lindenb.) J.C.Villarreal | Dominican Republic, Villarreal 864 (CONN) | ca. 800-1000 m. | HM056163 | HM067460 | HM067511 | HM067562 | JN559941 | HM163404 | --- |
| *Dendroceros difficilis Steph.* | Fiji, Von Konrat, 27407 | --- | HM056148 | HM067445 | HM067496 | HM067547 | JN559927 | --- | HM163344 |
| *Megaceros flagellaris Cargill 855 (CANB)* | Australia | -- | HM056151 | HM067448 | HM067499 | HM067550 | JN559929 | HM163392 | HM163347 |
| *Megaceros sp.* | Australia, Cargill & Fuhrer 535 (CANB) | --- | HM056150 | HM067447 | HM067498 | HM067549 | --- | HM163391 | HM163346 |
| *Phaeomegaceros fimbriatus (Gottsche) Duff et al.* | Costa Rica, Villarreal et al. 880 (CONN) | 3200 m. | HM056149 | HM067446 | HM067497 | HM067548 | JN559928 | HM163390 | HM163345 |

**Table S2.** *Nothoceros* divergence dates (in Ma) using a secondary calibration (modeled using a uniform prior) for the crown group of the genus *Nothoceros* and 2 different tree priors: birth death (A), and a Yule prior (B). The secondary calibration was obtained from Villarreal et al. (2015).

| **Two priors** | **Crown age of all**  ***N. aenigmaticus*** | **Crown age of**  ***N. aenigmaticus* exclusively from Mexico and US** |
| --- | --- | --- |
| **A-** birth death (bd) | **3.89** [1.03-7.76] | **0.79** [0.061-1.93] |
| **B-**  Yule prior | **4.35** [1.16-8.4] | **0.88** [0.06-2.13] |

**Table S3.** Summary statistics of the chlorotypes (cp) and mitotypes (mit) of *Nothoceros aenigmaticus* included in this study. The total number of samples (N) may differ from Table 1, because samples with one missing locus were excluded. N = sample size; G = number of haplotypes present in population; G_p_= number of private haplotypes; P = % of polymorphism, haplotypes present divided by total number of haplotypes; h = haploid genetic diversity defined by h=1- Σ*p^2^_i_*; uh = unbiased haploid genetic diversity defined by uh = (N/ (N-1)* h. uh corrects for sampling bias.

Populations and sexes: PR (Pigeon River, males), LPR (Little Pigeon River, males), BMP (Balsam Mountain Preserve, males), LTNR (Little TN River, females), HR (Hiwassee River, females), OC (Ocoee River, females), CR (Coosa River, females), ZM (Zempoala, females and males) and DM (los Diamantes, females).

|  | **Cp** | | | | | | **Mit** | | | | | |
| --- | --- | --- | --- | --- | --- | --- | --- | --- | --- | --- | --- | --- |
| **Populations** | **N** | **G** | **G_p_** | **P (%)** | **h** | **uh** | **N** | **G** | **G_p_** | **P (%)** | **h** | **uh** |
| **PR** | 42 | 4 | 2 | 16.6 | 0.328 | 0.336 | 44 | 5 | 2 | 12.8 | 0.417 | 0.427 |
| **LPR** | 59 | 6 | 4 | 25 | 0.496 | 0.504 | 60 | 9 | 6 | 23.0 | 0.517 | 0.526 |
| **BMP** | 9 | 1 | 0 | 8.3 | 0.198 | 0.222 | 10 | 2 | 2 | 5.1 | 0.48 | 0.533 |
| **LTNR** | 61 | 4 | 2 | 16.6 | 0.211 | 0.214 | 62 | 7 | 3 | 17.9 | 0.589 | 0.599 |
| **HR** | 36 | 3 | 0 | 8.3 | 0.156 | 0.160 | 36 | 4 | 0 | 10.2 | 0.477 | 0.49 |
| **OC** | 29 | 2 | 1 | 8.3 | 0.523 | 0.542 | 29 | 6 | 4 | 15.3 | 0.502 | 0.520 |
| **CR** | 8 | 1 | 0 | 4.1 | 0.00 | 0.00 | 8 | 2 | 0 | 5.1 | 0.375 | 0.429 |
| **ZM** | 18 | 7 | 7 | 29.1 | 0.796 | 0.843 | 11 | 9 | 9 | 23.0 | 0.86 | 0.945 |
| **DM** | 5 | 2 | 1 | 8.3 | 0.560 | 0.700 | 3 | 3 | 3 | 7.6 | 0.667 | 1.00 |
| Total | **267** | Total haplotypes = 24 | | | | | **263** | Total haplotypes = 39 | | | | |

**Table S4.** Summary statistics of microsatellite nuclear loci in *Nothoceros aenigmaticus* included in this study. N = sample size; Na = number of alleles present in population; Ne = number of effective alleles; I = Shannon's Information Index = -1* Sum (pi * Ln (pi)); h = diversity = 1 - Sum pi^2^; uh = unbiased haploid genetic diversity defined by uh = (N / (N-1))* h. Note: pi is the frequency of the ith allele for the population and Sum pi^2^ is the sum of the squared population allele frequencies.

Populations and sex: PR (Pigeon River, males), LPR (Little Pigeon River, males), BMP (Balsam Mountain Preserve, males), LTNR (Little TN River, females), HR (Hiwassee River, females), OC (Ocoee River, females), CR (Coosa River, females), ZM (Zempoala, females and males) and DM (Diamantes, females).

|  | **nuc35** | | | | | | **nuc38** | | | | | | **nuc42** | | | | | | **nuc52** | | | | | |
| --- | --- | --- | --- | --- | --- | --- | --- | --- | --- | --- | --- | --- | --- | --- | --- | --- | --- | --- | --- | --- | --- | --- | --- | --- |
|  | **N** | **Na** | **Ne** | **I** | **h** | **uh** | **N** | **Na** | **Ne** | **I** | **h** | **uh** | **N** | **Na** | **Ne** | **I** | **h** | **uh** | **N** | **Na** | **Ne** | **I** | **h** | **uh** |
| **PR** | 38 | 2 | 1.170 | 0.276 | 0.145 | 0.149 | 38 | 2 | 1.054 | 0.122 | 0.051 | 0.053 | 38 | 1 | 1.000 | 0.000 | 0.000 | 0.000 | 38 | 1 | 1.000 | 0.000 | 0.000 | 0.000 |
| **LPR** | 58 | 3 | 1.275 | 0.418 | 0.216 | 0.220 | 58 | 2 | 1.035 | 0.087 | 0.034 | 0.034 | 58 | 2 | 1.035 | 0.087 | 0.034 | 0.034 | 58 | 1 | 1.000 | 0.000 | 0.000 | 0.000 |
| **BMP** | 4 | 1 | 1.000 | 0.000 | 0.000 | 0.000 | 4 | 2 | 1.600 | 0.562 | 0.375 | 0.500 | 4 | 1 | 1.000 | 0.000 | 0.000 | 0.000 | 4 | 2 | 2.000 | 0.693 | 0.500 | 0.667 |
| **LTNR** | 61 | 2 | 1.033 | 0.084 | 0.032 | 0.033 | 61 | 2 | 1.140 | 0.242 | 0.123 | 0.125 | 61 | 1 | 1.000 | 0.000 | 0.000 | 0.000 | 61 | 1 | 1.000 | 0.000 | 0.000 | 0.000 |
| **HR** | 36 | 3 | 1.119 | 0.253 | 0.106 | 0.110 | 36 | 1 | 1.000 | 0.000 | 0.000 | 0.000 | 36 | 1 | 1.000 | 0.000 | 0.000 | 0.000 | 36 | 1 | 1.000 | 0.000 | 0.000 | 0.000 |
| **OR** | 27 | 3 | 1.255 | 0.420 | 0.203 | 0.211 | 27 | 2 | 1.077 | 0.158 | 0.071 | 0.074 | 27 | 1 | 1.000 | 0.000 | 0.000 | 0.000 | 27 | 1 | 1.000 | 0.000 | 0.000 | 0.000 |
| **CR** | 9 | 1 | 1.000 | 0.000 | 0.000 | 0.000 | 9 | 2 | 1.246 | 0.349 | 0.198 | 0.222 | 9 | 1 | 1.000 | 0.000 | 0.000 | 0.000 | 9 | 1 | 1.000 | 0.000 | 0.000 | 0.000 |
| **ZM** | 17 | 5 | 2.806 | 1.262 | 0.644 | 0.684 | 17 | 1 | 1.000 | 0.000 | 0.000 | 0.000 | 17 | 2 | 1.125 | 0.224 | 0.111 | 0.118 | 17 | 2 | 1.410 | 0.466 | 0.291 | 0.309 |
| **DM** | 5 | 2 | 1.923 | 0.673 | 0.480 | 0.600 | 5 | 1 | 1.000 | 0.000 | 0.000 | 0.000 | 5 | 1 | 1.000 | 0.000 | 0.000 | 0.000 | 5 | 1 | 1.000 | 0.000 | 0.000 | 0.000 |

**Table S5.** Pairwise _PT_ values (Fst analogous) among populations of *Nothoceros aenigmaticus* using mitotype data (chlorotype data show similar results, not shown).

Populations and sexes: PR (Pigeon River, males), LPR (Little Pigeon River, males), BMP (Balsam Mountain Preserve, males), LTNR (Little TN River, females), HR (Hiwassee River, females), OC (Ocoee River, females), CR (Coosa River, females), ZM (Zempoala, females and males) and DM (los Diamantes, females).

| **Statistic** | **LTNR** | **HR** | **OR** | **CR** | **PR** | **LPR** | **BM** | **ZM** | **DM** |
| --- | --- | --- | --- | --- | --- | --- | --- | --- | --- |
| **LTNR** | **--** | 0.013 | 0.434 | 0.450 | 0.479 | 0.437 | 0.422 | 0.271 | 0.302 |
| **HR** |  | -- | 0.496 | 0.528 | 0.543 | 0.489 | 0.495 | 0.334 | 0.393 |
| **OR** |  |  | -- | 0.000 | 0.532 | 0.477 | 0.475 | 0.293 | 0.363 |
| **CR** |  |  |  | -- | 0.573 | 0.501 | 0.515 | 0.307 | 0.388 |
| **PR** |  |  |  |  | -- | 0.04 | 0.541 | 0.387 | 0.455 |
| **LPR** |  |  |  |  |  | -- | 0.472 | 0.323 | 0.366 |
| **BM** |  |  |  |  |  |  | -- | 0.256 | 0.321 |
| **ZM** |  |  |  |  |  |  |  | -- | 0.035 |
| **DM** |  |  |  |  |  |  |  |  | -- |

**Table S6.** Summary statistics of clonal diversity, using all microsatellite data, in *N. aenigmaticus* using a threshold of 0/4/9 respectively. N = sample size; G = number of multilocus genotypes present in population; Eff = number of effective of multilocus genotype; div = Nei’s genetic diversity and eve = evenness (eff / G). The number of multilocus genotypes (G) per population divided by sample number (N) is also given. The values are estimated from multilocus genotypes excluding missing data (N = 248 total).

Populations and sexes: PR (Pigeon River, males), LPR (Little Pigeon River, males), BMP (Balsam Mountain Preserve, males), LTNR (Little TN River, females), HR (Hiwassee River, females), OC (Ocoee River, females), CR (Coosa River, females), ZM (Zempoala, females and males) and DM (los Diamantes, females).

**Threshold 0**

| **Population** | **size** | **num** | **eff** | **div** | **eve** | **G/N** |
| --- | --- | --- | --- | --- | --- | --- |
| Litle TN River | 62 | 13 | 4.169 | 0.773 | 0.321 | 0,20967742 |
| Hiwassee_ River | 36 | 8 | 2.817 | 0.663 | 0.352 | 0,22222222 |
| Occoee Nonotootla | 28 | 11 | 4.900 | 0.825 | 0.445 | 0,39285714 |
| Coosa River | 9 | 4 | 2.613 | 0.694 | 0.653 | 0,44444444 |
| Pigeon River | 44 | 15 | 4.190 | 0.779 | 0.279 | 0,34090909 |
| Little Pigeon River | 61 | 24 | 6.904 | 0.869 | 0.288 | 0,39344262 |
| Balsam Mountain Preserve | 10 | 3 | 2.273 | 0.622 | 0.758 | 0,3 |
| Zempoala (Mexico) | 19 | 11 | 3.967 | 0.789 | 0.361 | 0,57894737 |
| Diamantes (Mexico) | 5 | 4 | 3.571 | 0.900 | 0.720 | 0.8 |

**Threshold 4**

| **Population** | **size** | **num** | **eff** | **div** | **eve** | **G/N** |
| --- | --- | --- | --- | --- | --- | --- |
| Litle TN River | 62 | 5 | 1.263 | 0.212 | 0.253 | 0,08064516 |
| Hiwassee_ River | 36 | 4 | 1.187 | 0.162 | 0.297 | 0,11111111 |
| Occoee Nonotootla | 28 | 6 | 1.867 | 0.481 | 0.311 | 0,21428571 |
| Coosa River | 9 | 3 | 1.588 | 0.417 | 0.529 | 0,33333333 |
| Pigeon River | 44 | 10 | 2.482 | 0.611 | 0.248 | 0,22727273 |
| Little Pigeon River | 61 | 11 | 1.807 | 0.454 | 0.164 | 0,18032787 |
| Balsam Mountain Preserve | 10 | 3 | 2.273 | 0.622 | 0.758 | 0,3 |
| Zempoala (Mexico) | 19 | 9 | 3.722 | 0.772 | 0.414 | 0,47368421 |
| Diamantes (Mexico) | 5 | 4 | 3.571 | 0.900 | 0.720 | 0.8 |

**Threshold 9**

| **Population** | **size** | **num** | **eff** | **div** | **eve** | **G/N** |
| --- | --- | --- | --- | --- | --- | --- |
| Litle TN River | 62 | 3 | 1.067 | 0.064 | 0.356 | 0,0483871 |
| Hiwassee_ River | 36 | 2 | 1.057 | 0.056 | 0.529 | 0,05555556 |
| Occoee Nonotootla | 28 | 6 | 1.867 | 0.481 | 0.311 | 0,21428571 |
| Coosa River | 9 | 2 | 1.246 | 0.222 | 0.623 | 0,22222222 |
| Pigeon River | 44 | 4 | 1.460 | 0.322 | 0.365 | 0,09090909 |
| Little Pigeon River | 61 | 6 | 1.271 | 0.217 | 0.212 | 0,09836066 |
| Balsam Mountain Preserve | 10 | 3 | 2.273 | 0.622 | 0.758 | 0,3 |
| Zempoala (Mexico) | 19 | 9 | 3.722 | 0.772 | 0.414 | 0,47368421 |
| | Diamantes (Mexico) | 5 | 4 | 3.571 | 0.900 | 0.720 | 0.8 | | --- | --- | --- | --- | --- | --- | --- | | 5 | 3 | 2.778 | 0.800 | 0.926 | 0.6 |

**Table S7.** K-Means clustering and population assignment using all microsatellite data and different thresholds (0/4/9 respectively).

**Threshold 0**

| **k** | **SSD(T)** | **SSD(AC)** | **SSD(WC)** | **r-squared** | **pseudo-F** | **AIC** | **BIC** |
| --- | --- | --- | --- | --- | --- | --- | --- |
| 3 | 1286.371 | 804.937 | 481.434 | 0.868 | 19.688 | 49.876 | 0.657 |
| 4 | 1286.371 | 867.925 | 418.446 | 0.936 | 24.243 | 45.588 | 0.719 |
| 5 | 1286.371 | 888.008 | 398.363 | 0.957 | 22.433 | 44.092 | 0.691 |
| 6 | 1286.371 | 907.555 | 378.816 | 0.978 | 27.176 | 40.162 | 0.729 |
| 7&* | 1286.371 | 920.478 | 365.893 | 0.992 | 43.128 | 33.039 | 7&* |
|  | | | | **K=7 Little TN River/Hiwassee**, **Ocoee**/**Coosa River**, **Pigeon,Little Pigeon River, BMP Preserve, Zempoala, Diamantes.** | | | |
| * Best clustering according to Calinski & Harabasz' pseudo-F: k = 7  & Best clustering according to Bayesian Information Criterion: k = 7 | | | |  |  |  |  |

**Threshold 4**

| **k** | **SSD(T)** | **SSD(AC)** | **SSD(WC)** | **r-squared** | **pseudo-F** | **AIC** | **BIC** |
| --- | --- | --- | --- | --- | --- | --- | --- |
| 3 | 36.325 | 20.973 | 15.352 | 0.577 | 4.099 | 15.606 | 31.173 |
| 4 | 36.325 | 27.939 | 8.387 | 0.769 | 5.552 | 17.365 | 27.929 |
| 5 | 36.325 | 32.694 | 3.631 | 0.900 | 9.004 | 21.831 | 22.592 |
| 6 | 36.325 | 35.494 | 0.831 | 0.977 | 25.620 | 32.561 | 11.520 |
| 7&* | 36.325 | 35.922 | 0.404 | 0.989 | 29.669 | 98.059 | 7.214 |
|  | | | | **K=7 Little TN River/Hiwassee**, **Ocoee**/**Coosa River**, **Pigeon,Little Pigeon River, BMP Preserve, Zempoala, Diamantes.** | | | |
| * Best clustering according to Calinski & Harbasz' pseudo-F: k = 7  & Best clustering according to Bayesian Information Criterion: k = 7 | | | |  |  |  |  |

**Threshold 9**

| **k** | **SSD(T)** | **SSD(AC)** | **SSD(WC)** | **r-squared** | **pseudo-F** | **AIC** | **BIC** |
| --- | --- | --- | --- | --- | --- | --- | --- |
| 3 | 36.325 | 20.973 | 15.352 | 0.577 | 4.099 | 15.606 | 31.173 |
| 4 | 36.325 | 27.939 | 8.387 | 0.769 | 5.552 | 17.365 | 27.929 |
| 5 | 36.325 | 32.694 | 3.631 | 0.900 | 9.004 | 21.831 | 22.592 |
| 6 | 36.325 | 35.494 | 0.831 | 0.977 | 25.620 | 32.561 | 11.520 |
| 7&* | 36.325 | 35.922 | 0.404 | 0.989 | 29.669 | 98.059 | 7.214 |
|  | | | | **K=7 Little TN River/Hiwassee**, **Ocoee**/**Coosa River**, **Pigeon,Little Pigeon River, BMP Preserve, Zempoala, Diamantes.** | | | |
| * Best clustering according to Calinski & Harabasz' pseudo-F: k = 7  & Best clustering according to Bayesian Information Criterion: k = 7 | | | |  |  |  |  |

**Table S8.** Summary of watersheds statistics of *Nothoceros aenigmaticus*. Population statistics have been split into statistics for polymorphic loci only and for both polymorphic and fixed loci. Filters applied were *–max-het-obs 0 --min-maf 0.05 –r 0.5*. Statistics include the total average number of individuals genotyped at each locus (N), the number of alleles unique to each population (Private), mean nucleotide diversity (π), the total number of sites for each populations (sites), the number of sites across all populations that are variant (variant sites), the number of sites inside each population that are variant (polymorphic sites) and the percentage of polymorphic sites (PPL %).

Populations and sexes: PR (Pigeon River, males), LPR (Little Pigeon River, males), LTNR (Little TN River, females), HR (Hiwassee River, females), OC (Ocoee River, females), CR (Coosa River, females) and ZM (Zempoala, females and males.

| **Watersheds** | **Variant positions** | | | **Variant and fixed positions** | | | | | | |
| --- | --- | --- | --- | --- | --- | --- | --- | --- | --- | --- |
|  | **N** | **Private** | **π** | **N** | **Private** | **Sites** | **Variant sites** | **Polymorphic sites** | **PPL %** | **π** |
| **PR** | 15 | 8 | 0.00175 | 15 | 8 | 313286 | 688 | 6 | 0.00192 | 0.00000 |
| **LPR** | 17 | 3 | 0.00368 | 17 | 3 | 272432 | 665 | 10 | 0.00367 | 0.00001 |
| **LTNR** | 14 | 3 | 0.00208 | 14 | 3 | 302856 | 692 | 4 | 0.00132 | 0.00000 |
| **HR** | 11 | 2 | 0.00189 | 11 | 2 | 320335 | 717 | 5 | 0.00156 | 0.00000 |
| **OR** | 2 | 6 | 0.00000 | 2 | 6 | 283678 | 721 | 0 | 0.00000 | 0.00000 |
| **CR** | 5 | 2 | 0.00172 | 5 | 2 | 267585 | 694 | 3 | 0.00112 | 0.00000 |
| **ZM** | 5 | 307 | 0.05436 | 5 | 307 | 322859 | 720 | 93 | 0.02881 | 0.00012 |

**Table S9.** Summary statistics of clonal diversity in *N. aenigmaticus*, using SNP data, using a threshold of 0 and clones specific to each population. N = sample size; G = number of multilocus genotypes present in population; G = number of effective of multilocus genotypes; div = Nei’s genetic diversity and eve = evenness (eff / G). The number of multilocus genotypes (G) per population divided by sample number (N) is also given.

Populations and sexes: PR (Pigeon River, males), LPR (Little Pigeon River, males), LTNR (Little TN River, females), HR (Hiwassee River, females), OC (Ocoee River, females), CR (Coosa River, females) and ZM (Zempoala, females and males.

| **Population** | **N** | **G** | **Eff** | **div** | **Eve** | **G/N** |
| --- | --- | --- | --- | --- | --- | --- |
| **PR** | 17 | 1 | 1 | 0 | 1 | 0.05 |
| **LPR** | 24 | 1 | 1 | 0 | 0.692 | 0.04 |
| **LTNR** | 17 | 1 | 1 | 0 | 1 | 0.05 |
| **HR** | 14 | 2 | 1.15 | 0.143 | 0.576 | 0.07 |
| **OR** | 2 | 1 | 1 | 0 | 1 | 0.5 |
| **CR** | 6 | 2 | 4.5 | 0.33 | 0.9 | 0.33 |
| **ZM** | 6 | 5 | 4.5 | 0.933 | 1 | 0.83 |

**Table S10.** Fst values (G-statistics) using SNP data estimated from all pairs of populations based 1000 permutations (estimated in Genodive).

Populations and sexes: PR (Pigeon River, males), LPR (Little Pigeon River, males), LTNR (Little TN River, females), HR (Hiwassee River, females), OC (Ocoee River, females), CR (Coosa River, females) and ZM (Zempoala, females and males.

| **Populations** | **LTNR** | **HR** | **LPR** | **ZM** | **OR** | **CR** | **PR** |
| --- | --- | --- | --- | --- | --- | --- | --- |
| **LTNR** | -- |  |  |  |  |  |  |
| **HR** | 0.363 | -- |  |  |  |  |  |
| **LPR** | 0.993 | 0.993 | -- |  |  |  |  |
| **ZM** | 0.981 | 0.979 | 0.98 | -- |  |  |  |
| **OR** | 0.995 | 0.996 | 0.991 | 0.941 | -- |  |  |
| **CR** | 0.996 | 0.997 | 0.991 | 0.961 | -0.304 | -- |  |
| **PR** | 0.996 | 0.996 | **0.286** | 0.981 | 0.996 | 0.996 | -- |
|  |  |  |  |  |  |  |  |
| **- p-values** |  |  |  |  |  |  |  |
| **p-values** | **LTNR** | **HR** | **LPR** | **ZM** | **OR** | **CR** | **PR** |
| **LTNR** | -- | 0.001 | 0.001 | 0.001 | 0.009 | 0.001 | 0.001 |
| **HR** | 0.001 | -- | 0.001 | 0.001 | 0.007 | 0.001 | 0.001 |
| **LPR** | 0.001 | 0.001 | -- | 0.001 | 0.004 | 0.001 | 0.001 |
| **ZM** | 0.001 | 0.001 | 0.001 | -- | 0.032 | 0.002 | 0.001 |
| **OR** | 0.009 | 0.007 | 0.004 | 0.032 | -- | 1 | 0.008 |
| **CR** | 0.001 | 0.001 | 0.001 | 0.002 | 1 | -- | 0.001 |
| **PR** | 0.001 | 0.001 | 0.001 | 0.001 | 0.008 | 0.001 | -- |

**Table S11.** K-Means clustering and population assignment using SNP data.

| **k** | **SSD(T)** | **SSD(AC)** | **SSD(WC)** | **r-squared** | **pseudo-F** | **AIC** | **BIC** |
| --- | --- | --- | --- | --- | --- | --- | --- |
| 2 | 1639.638 | 609.57 | 1030.069 | 0.372 | 2.959 | 41.94 | 52.453 |
| 3 | 1639.638 | 1172.913 | 466.725 | 0.715 | 5.026 | 43.399 | 48.858 |
| 4 | 1639.638 | 1637.016 | 2.623 | 0.998 | 624.18 | 21.128 | 14.533 |
| 5& | 1639.638 | 1638.015 | 1.623 | 0.999 | 504.563 | 59.769 | 13.12 |
|  |  |  |  |  |  |  |  |
|  | | | | **K=5 Little TN River/Hiwassee**, **Ocoee**, **Coosa River**, **Pigeon/Little Pigeon River, Zempoala.** | | | |
| & Best clustering according to Calinski & Harabasz' pseudo-F and Bayesian Information Criterion: k = 5 | | | |  |  |  |  |

**Table S12.** Current and inferred population membership for all individuals using SNP data, as well as ln(likelihood) for population membership for all individuals and all populations with at least 5 individuals: all in gray. Likelihood ratio thresholds for all 7 populations based on the Monte Carlo test, with an alpha of 0.00200. 100 replicated datasets, with a total of 7,099 resampled individuals.

Populations and sexes: PR (Pigeon River, males), LPR (Little Pigeon River, males), LTNR (Little TN River, females), HR (Hiwassee River, females), OC (Ocoee River, females), CR (Coosa River, females) and ZM (Zempoala, females and males.

| **Individual** | **Current** | **Inferred** | **Lik_max** | **Lik_home** | **Lik_ratio** |
| --- | --- | --- | --- | --- | --- |
| Ind001 | LTNR | LTNR | -3.894 | -3.894 | 0 |
| Ind002 | LTNR | LTNR | -4.826 | -4.826 | 0 |
| Ind003 | LTNR | HR | -2.589 | -4.062 | 2.945 |
| Ind004 | LTNR | HR | -1.624 | -1.699 | 0.15 |
| Ind005 | LTNR | HR | -4.076 | -4.959 | 1.765 |
| Ind006 | LTNR | LTNR | -4.15 | -4.15 | 0 |
| Ind007 | LTNR | LTNR | -4.245 | -4.245 | 0 |
| Ind008 | LTNR | LTNR | -1.117 | -1.117 | 0 |
| Ind009 | LTNR | HR | -3.971 | -4.853 | 1.765 |
| Ind010 | LTNR | LTNR | -3.91 | -3.91 | 0 |
| Ind011 | LTNR | LTNR | -4.17 | -4.17 | 0 |
| Ind012 | LTNR | LTNR | -4.195 | -4.195 | 0 |
| Ind013 | LTNR | LTNR | -4.926 | -4.926 | 0 |
| Ind014 | LTNR | HR | -3.294 | -4.177 | 1.765 |
| Ind015 | LTNR | HR | -3.478 | nan | nan |
| Ind016 | LTNR | LTNR | -8.105 | -8.105 | 0 |
| Ind017 | LTNR | LTNR | -4.751 | -4.751 | 0 |
| Ind018 | HR | HR | -2.653 | -2.653 | 0 |
| Ind019 | HR | LTNR | -10.136 | nan | nan |
| Ind020 | HR | HR | -4.202 | -4.202 | 0 |
| Ind021 | HR | HR | -2.952 | -2.952 | 0 |
| Ind022 | HR | HR | -4.514 | -4.514 | 0 |
| Ind023 | HR | HR | -4.499 | -4.499 | 0 |
| Ind024 | HR | HR | -4.514 | -4.514 | 0 |
| Ind025 | HR | HR | -2.887 | -2.887 | 0 |
| Ind026 | HR | HR | -4.121 | -4.121 | 0 |
| Ind027 | HR | HR | -1.699 | -1.699 | 0 |
| Ind028 | HR | HR | -3.113 | -3.113 | 0 |
| Ind029 | HR | HR | -3.625 | -3.625 | 0 |
| Ind030 | HR | LTNR | -8.621 | -9.059 | 0.875 |
| Ind031 | HR | HR | -12.04 | -12.04 | 0 |
| Ind032 | LPR | LPR | -7.296 | -7.296 | 0 |
| Ind033 | LPR | PR | -9.818 | -10.417 | 1.199 |
| Ind034 | LPR | PR | -4.885 | -4.91 | 0.05 |
| Ind035 | LPR | LPR | -6.819 | -6.819 | 0 |
| Ind036 | LPR | PR | -9.918 | -10.497 | 1.159 |
| Ind037 | LPR | PR | -4.379 | -4.975 | 1.193 |
| Ind038 | LPR | PR | -4.304 | -4.883 | 1.159 |
| Ind039 | LPR | LPR | -6.346 | -6.346 | 0 |
| Ind040 | LPR | PR | -3.065 | -8.561 | 10.993 |
| Ind041 | LPR | PR | -2.208 | -3.393 | 2.371 |
| Ind042 | LPR | PR | -1.421 | -1.871 | 0.899 |
| Ind043 | LPR | PR | -1.722 | -3.333 | 3.223 |
| Ind044 | LPR | LPR | -3.377 | -3.377 | 0 |
| Ind045 | LPR | LPR | -1.712 | -1.712 | 0 |
| Ind046 | LPR | LPR | -1.176 | -1.176 | 0 |
| Ind047 | LPR | LPR | -6.967 | -6.967 | 0 |
| Ind048 | LPR | LPR | -6.061 | -6.061 | 0 |
| Ind049 | LPR | LPR | -6.035 | -6.035 | 0 |
| Ind050 | LPR | LPR | -6.695 | -6.695 | 0 |
| Ind051 | LPR | LPR | -7.113 | -7.113 | 0 |
| Ind052 | LPR | PR | -8.493 | -9.904 | 2.823 |
| Ind053 | LPR | LPR | -12.392 | -12.392 | 0 |
| Ind054 | LPR | LPR | -6.944 | -6.944 | 0 |
| Ind055 | LPR | PR | -8.713 | -10.679 | 3.932 |
| Ind056 | ZM | CR | -2020.129 | nan | nan |
| Ind057 | ZM | ZM | -77.756 | -77.756 | 0 |
| Ind058 | ZM | CR | -1698.512 | nan | nan |
| Ind059 | ZM | ZM | -47.067 | -47.067 | 0 |
| Ind060 | ZM | CR | -2219.419 | nan | nan |
| Ind061 | ZM | CR | -1646.91 | nan | nan |
| Ind062 | ONR | CR | -3.445 | --- | --- |
| Ind063 | ONR | CR | -3.305 | --- | --- |
| Ind064 | CR | CR | -7.384 | -7.384 | 0 |
| Ind065 | CR | CR | -3.536 | -3.536 | 0 |
| Ind066 | CR | CR | -2.083 | -2.083 | 0 |
| Ind067 | CR | CR | -3.236 | -3.236 | 0 |
| Ind068 | CR | CR | -2.965 | -2.965 | 0 |
| Ind069 | CR | CR | -3.421 | -3.421 | 0 |
| Ind070 | PR | LPR | -9.47 | -10.324 | 1.708 |
| Ind071 | PR | LPR | -9.713 | -10.336 | 1.245 |
| Ind072 | PR | LPR | -4.973 | -5.336 | 0.725 |
| Ind073 | PR | PR | -4.042 | -4.042 | 0 |
| Ind074 | PR | PR | -4.062 | -4.062 | 0 |
| Ind075 | PR | PR | -3.681 | -3.681 | 0 |
| Ind076 | PR | PR | -4.077 | -4.077 | 0 |
| Ind077 | PR | PR | -8.272 | -8.272 | 0 |
| Ind078 | PR | PR | -3.891 | -3.891 | 0 |
| Ind079 | PR | PR | -3.926 | -3.926 | 0 |
| Ind080 | PR | PR | -3.651 | -3.651 | 0 |
| Ind081 | PR | PR | -4.062 | -4.062 | 0 |
| Ind082 | PR | PR | -3.881 | -3.881 | 0 |
| Ind083 | PR | PR | -3.255 | -3.255 | 0 |
| Ind084 | PR | PR | -1.902 | -1.902 | 0 |
| Ind085 | PR | PR | -1.077 | -1.077 | 0 |
| Ind086 | PR | LPR | -5.308 | -10.302 | 9.987 |

**Table S13.** Spatial genetic structure parameters of *Nothoceros aenigmaticus* from US for contiguous watershed. F_ij_ average kinship coefficient between individuals from the significant distance classes (0,25 and 1 km in PR and LPR, 1 km in LTNR and HR, and 5, 10 and 20 km in OC and CR km, see Figure S3), b_log_ the regression slope of F_ij_ on log spatial distance are given with SD.

|  |  | **F_ij_** | **b_log_** | **SD** |
| --- | --- | --- | --- | --- |
| **PR and LPR** (0.25 and 1 km) | **Ramets** | 0.2631 (0.25)  0.3170 (1) | -0.0967 | 0.0069 |
|  | **Genets** | 0.1494 (0.25)  0.1578 (1) | -0.0323 | 0.0050 |
| **LTNR and HR** (1 km) | **Ramets** | 0.5977 | -0.0791 | 0.0085 |
|  | **Genets** | 0.3755 | -0.0593 | 0.0071 |
| **OC and CR**  (5, 10 and 20 km) | **Ramets** | 0.3115 (5)  0.2328 (10)  0.2473 (20) | -0.1717 | 0.0166 |
|  | **Genets** | 0.1920 (5)  0.1478 (20) | -0.1059 | 0.0175 |
