# Supplementary material for: Population Genomics and Phylogeography of a Clonal Bryophyte With Spatially Separated Sexes and Extreme Sex Ratios
Source: Front Plant Sci. 2020 May 8;11:495. doi: 10.3389/fpls.2020.00495 (PMC7226906; doi:10.3389/fpls.2020.00495)
Supplement: Supplementary file 2 [file Data_Sheet_2.docx]

**SUPPLEMENT**

**Supplement Figure 1.** **A**. Maximum likelihood phylogeny of *Nothoceros* *aenigmaticus* and outgroup taxa based on seven nuclear, plastid, and mitochondrial loci; ML bootstrap values (as circles on the branches). We highlighted *Nothoceros aenigmaticus* from Paramos (in red) and *Nothoceros aenigmaticus* from Mexico and the US (in green). **B.** *Nothoceros* divergence dates (in Mya) using a secondary calibration (modeled using a uniform prior) for the crown group of the genus *Nothoceros* and a birth death tree prior. We selected some clades to show the high posterior density (HPD, blue horizontal bars).The chronogram shows the recent origin of *N. aenigmaticus* from high elevation tropical habitats above 3,000 mts. (Paramos) (ca. 3-4 Mya on average, in red) and the US/Mexico *N. aenigmaticus* (0.79-.88 Mya on average, in green). See also ­­the inset graph of the HPD of the two clades. The nodes with >0.95 of posterior probabilities are denoted by a red circle and the turquoise bar represents the formation of Andean paramos.

**Supplement Figure 2.** Chlorotype and mitotype diversity based on microsatellites of *Nothoceros aenigmaticus*. The pies above the diagonal line represent the mitotypes and below the line, the chlorotypes, pie charts were drawn using https://www.meta-chart.com. Watersheds sharing at least one chlorotype or mitotype are color-coded. The Pigeon and Little Pigeon River (blue oval); Balsam Mountain Preserve (blue oval); Little Tennessee and Hiwassee River (reddish oval) and Ocoee and Coosa River (orange oval). The two localities draining into the Coosa River are circled within the Ocoee/Coosa River cluster (orange oval). Black dots represent sampling sites for female plants and blue dots for male plants. The Little Pigeon (9), Little TN (7) and Ocoee Rivers (6) show the highest number of mitotypes amongst Southern Appalachian populations.

**Supplement Figure 3.** Spatial genetic structure correlograms of *Nothoceros aenigmaticus* from US for contiguous watersheds (LPR and PR, LTNR and HR, ONR and CR) at ramets and genets’ level. Graphs show mean kinship coefficient (F_ij_) between pairs of ramets or genets over eight distance classes. The 95%-CIs show the lower and the upper 95% confidence interval values. Populations and sex: PR (Pigeon River, males), LPR (Little Pigeon River, males), LTNR (Little TN River, females), HR (Hiwassee River, females), OC (Ocoee River, females), CR (Coosa River, females).
