# Supplementary figures and images for: Population Genomics and Phylogeography of a Clonal Bryophyte With Spatially Separated Sexes and Extreme Sex Ratios
Source: Front Plant Sci. 2020 May 8;11:495. doi: 10.3389/fpls.2020.00495 (PMC7226906; doi:10.3389/fpls.2020.00495)

**A**

Bootstrap 500 replicates

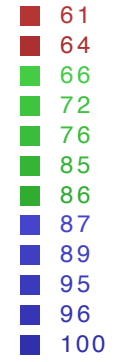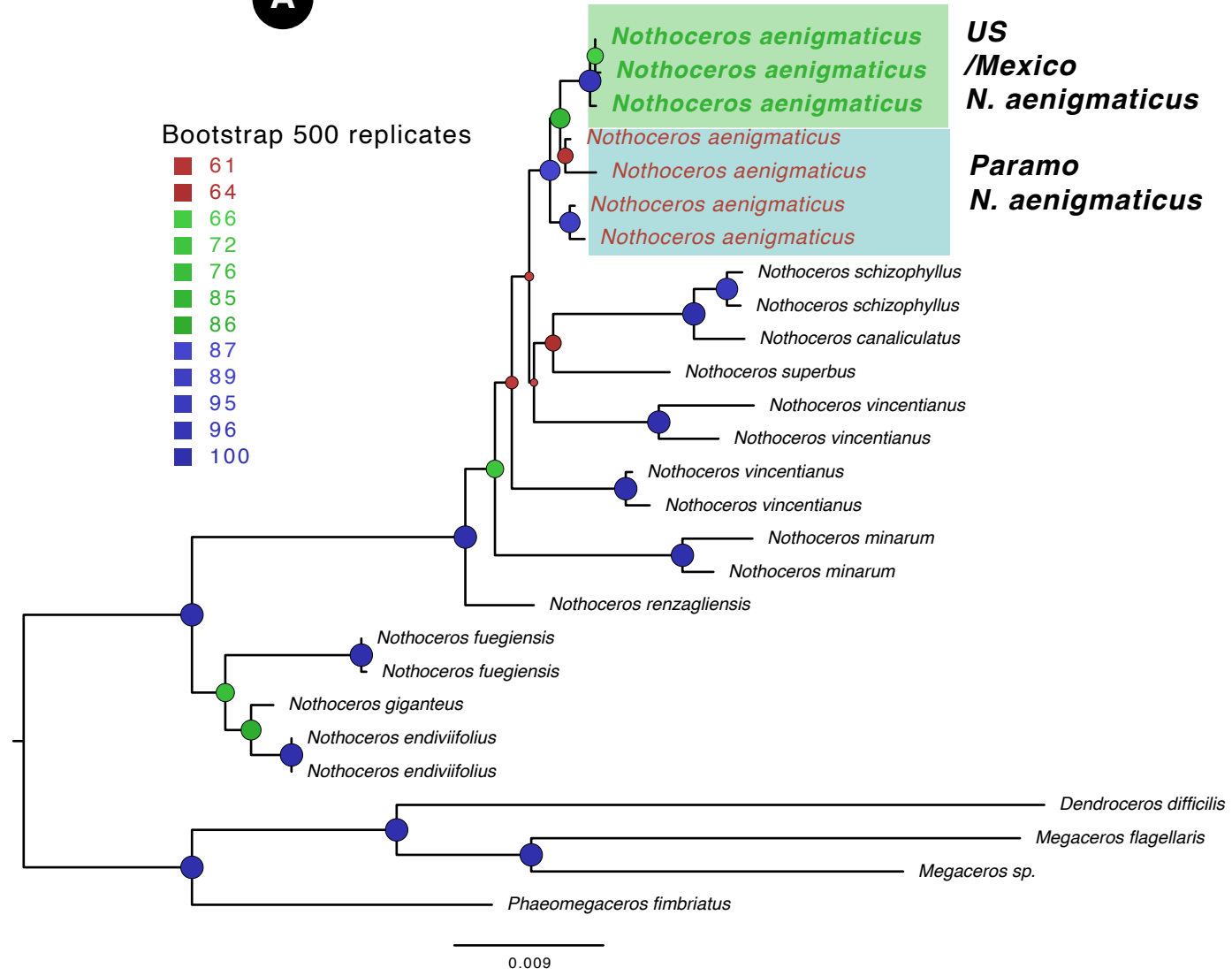

**B**

posterior  
1

0.5925

Paramo formation

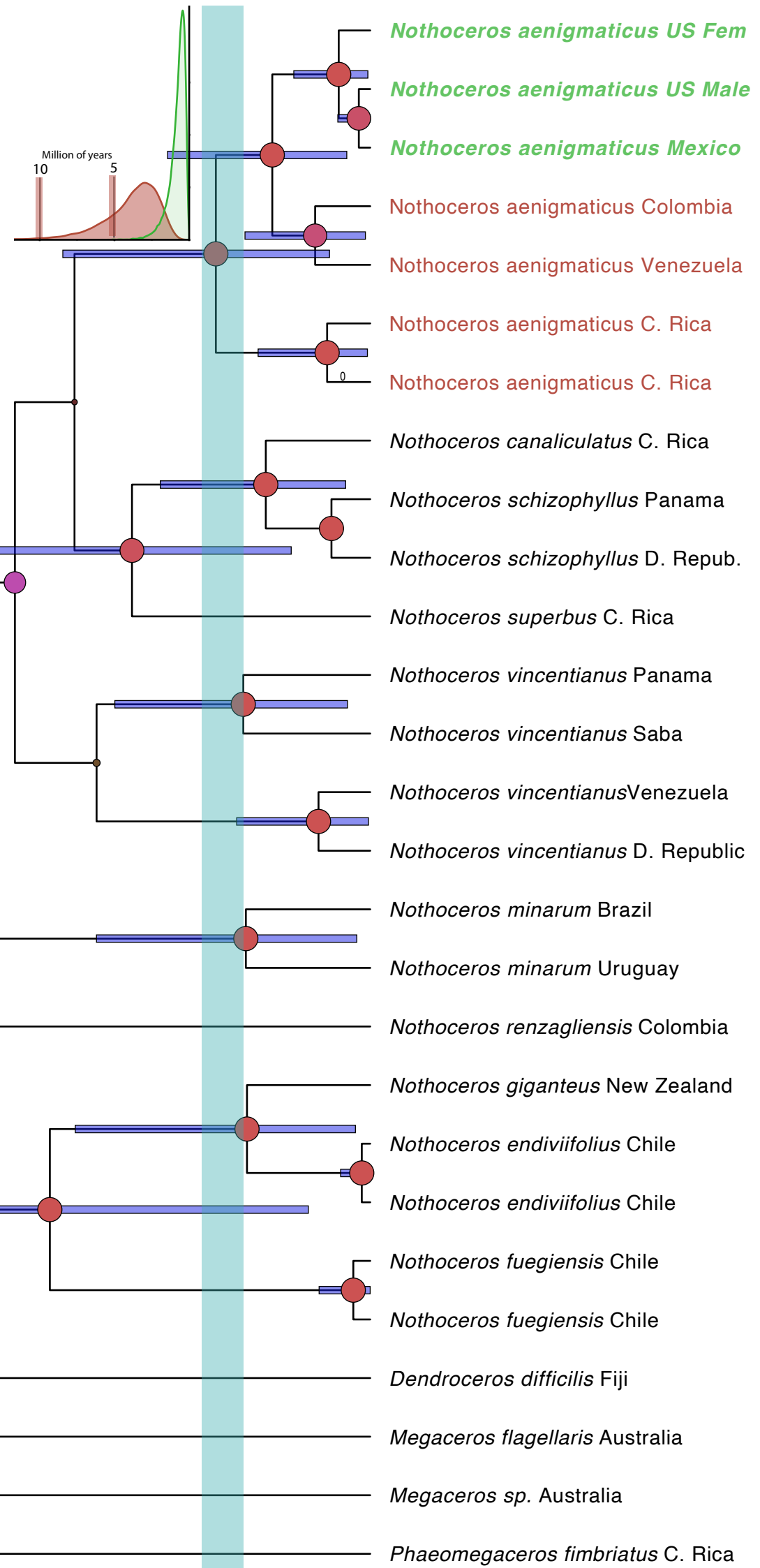

Million of years ago 40 30 20 10 0

Supplement: Supplementary file 3 [file Image_1.pdf]

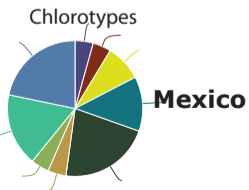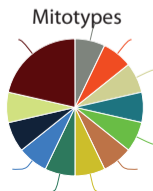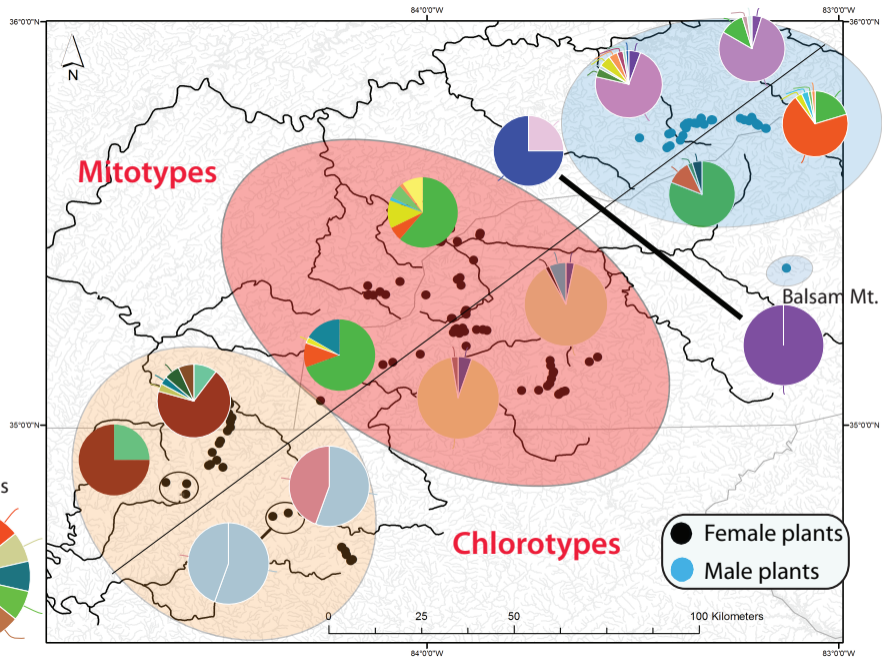

Supplement: Supplementary file 4 [file Image_2.pdf]

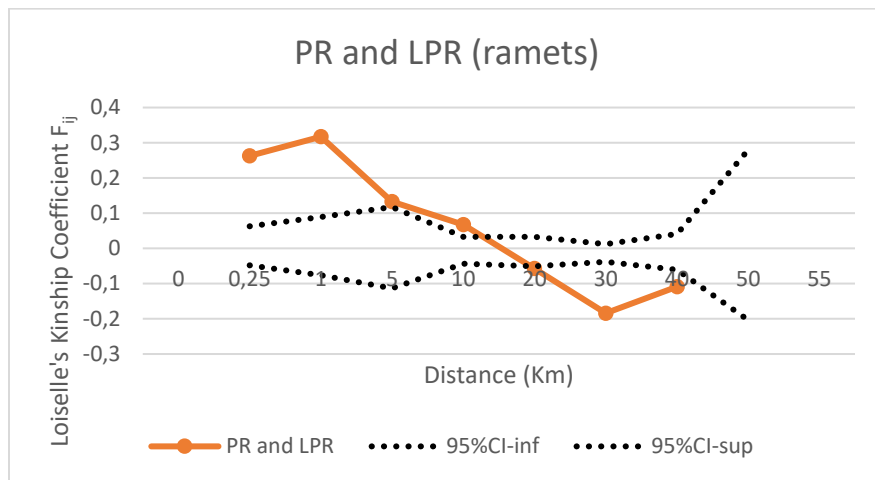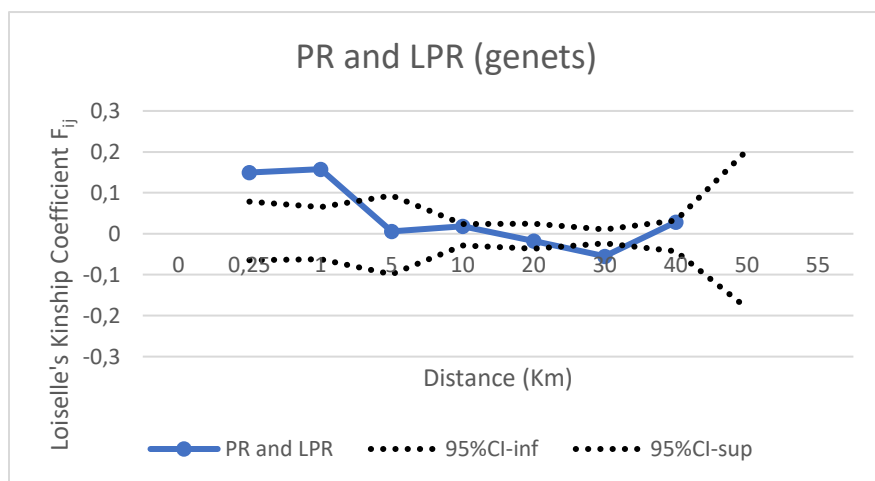

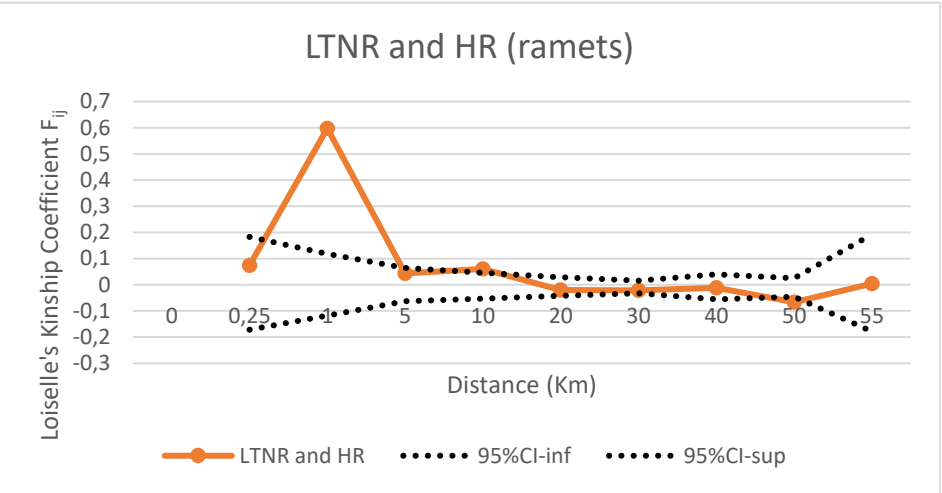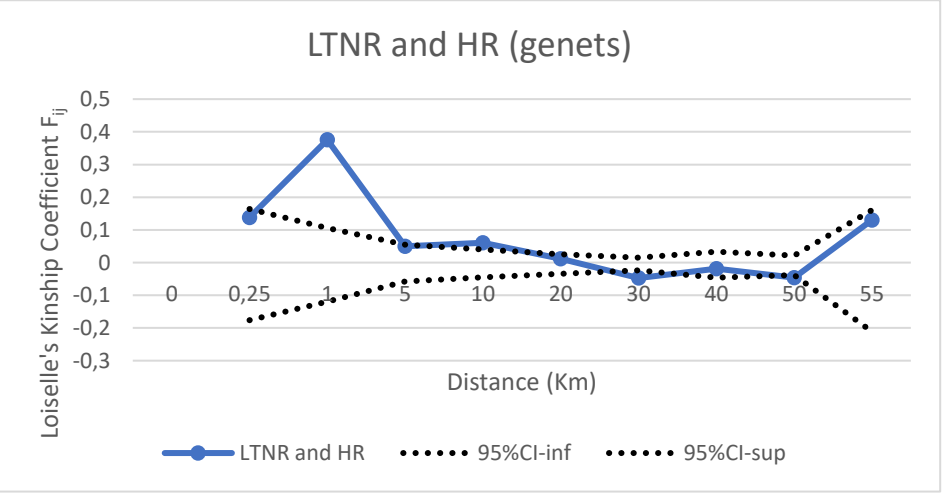

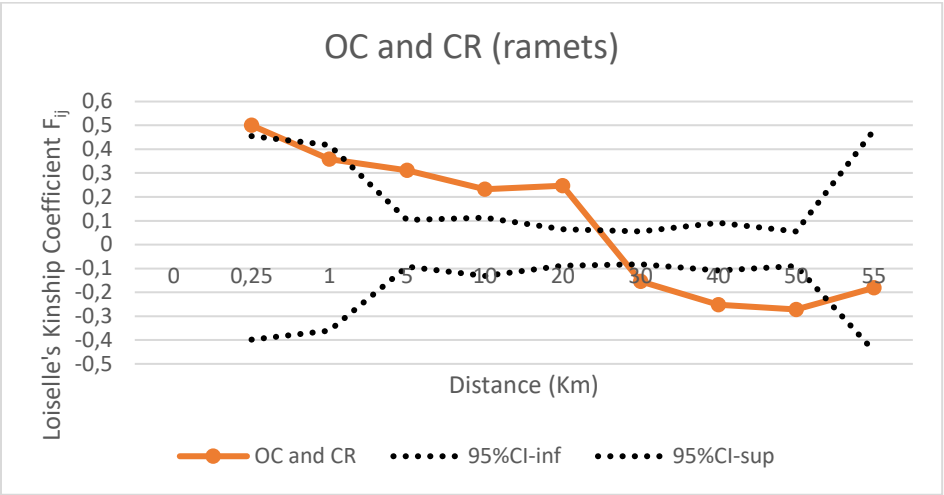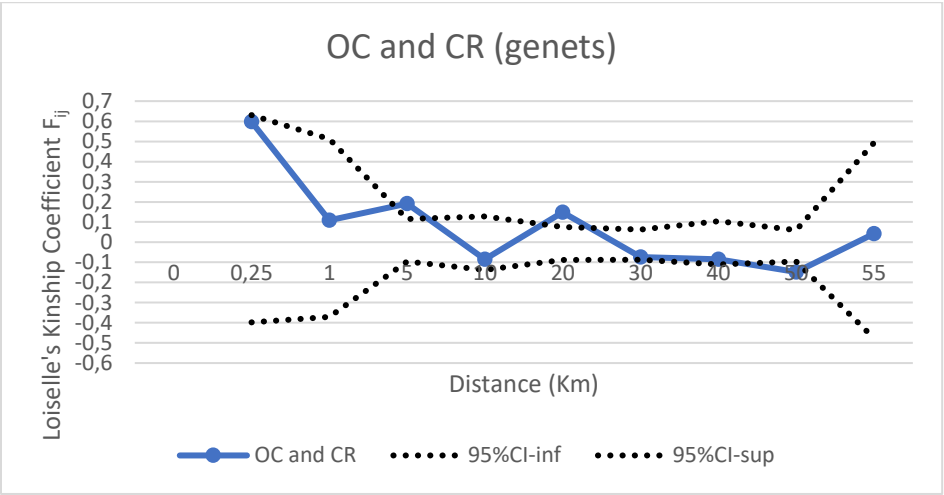

Supplement: Supplementary file 5 [file Image_3.pdf]
